# The Evolution of Data-Driven Management Zone Delineation: A Systematic Review

**DOI:** 10.3390/s26103249

**Published:** 2026-05-20

**Authors:** Roghayeh Heidari, Reza Khanmohammadi, Faramarz F. Samavati

**Affiliations:** 1 Department of Computer Science, University of Calgary, Calgary, AB T2N 1N4, Canada; samavati@ucalgary.ca; 2 DeepTern AI Inc., Calgary, AB T2L 1Y3, Canada; reza@deeptern.ai

**Keywords:** precision agriculture, variable rate technology, management zone delineation, data fusion, clustering algorithms, spatial modeling, geostatistics

## Abstract

By partitioning agricultural fields into units with similar yield-limiting factors, Management Zone (MZ) delineation provides the spatial basis for variable-rate application of inputs such as nitrogen, seed, and irrigation. To evaluate the operational implementation of MZ methodologies, this paper analyzes 137 peer-reviewed papers published between 2000 and 2025, extracting data on agronomic contexts, sensing inputs, computational workflows, and validation strategies. Our analysis reveals a clear methodological shift: while early studies relied heavily on data such as soil properties, recent literature is dominated by multisource data fusion that combines static soil proxies (e.g., apparent electrical conductivity) with dynamic remote sensing vegetation indices. Methodologically, the literature relies heavily on similarity-based clustering, specifically fuzzy c-means and k-means, often applied to raw spatial grids or Principal Component Analysis (PCA) transformations. Although machine learning and optimization-based approaches have increased in recent years, rigorous agronomic and economic validation remains limited, while internal cluster validity indices (e.g., FPI, NCE) and inferential statistical tests (e.g., ANOVA) are widely used to assess delineated zones, only 13 of the reviewed papers explicitly evaluated the economic or environmental net returns of the delineated zones. To transition MZ delineation from a classification problem to an operational decision-support tool, the current literature suggests a need to shift validation efforts away from internal clustering metrics toward multi-year yield stability assessments and direct economic cost–benefit analyses.

## 1. Introduction

Agricultural fields commonly exhibit spatial variability in soil properties, topography, and crop performance. Managing inputs such as water, fertilizers, and agrochemicals uniformly across those heterogeneous environments often results in suboptimal resource use [1]. Precision agriculture addresses this challenge by enabling site-specific management, in which agronomic inputs are adjusted to the localized conditions [2]. Such technologies may be implemented through real-time, sensor-based systems that adjust inputs on the fly, or through map-based approaches that rely on predefined spatial units [3,4]. Management zones (MZs) are central to the latter approach, allowing fields to be subdivided into relatively homogeneous sub-areas that can be managed as distinct units. By tailoring resources to localized soil and crop conditions, MZs aim to enhance resource use efficiency, yield stability, and sustainability [1].

By aggregating spatial variability into discrete field units, MZs enable the application of site-specific prescriptions that align with the physical maneuvering constraints of standard variable-rate machinery [2,5]. However, the effectiveness of this approach depends critically on the delineation of zones that capture stable and agronomically meaningful patterns of crop response [6].

The challenge of identifying such patterns has driven extensive research to characterize within-field variability. Early MZ papers relied on soil sampling [7], yield data [8], and apparent electrical conductivity ($EC_{a}$) mapping [9]. Subsequent advancements introduced digital elevation models (DEMs) and terrain features [10,11], multispectral and hyperspectral satellite imagery [12], imagery acquired from Unmanned Aerial Vehicles (UAVs) [13], proximal canopy sensors [14], and in-field Internet-of-Things (IoT) networks [15]. These data differ markedly in temporal stability: soil, $EC_{a}$, and terrain features represent long-term controls, whereas vegetation indices (VIs), extracted from remote sensing imagery, and yield maps reflect transient or seasonal processes. As a result, recent operational MZ delineation workflows often integrate multiple data sources to balance static and dynamic drivers of variability [16].

In addition, the choice of data sources and computational methods for MZ delineation often varies with the agronomic system: for instance, rainfed cereal systems predominantly rely on stable soil proxies such as $EC_{a}$ [17], whereas perennial orchard systems frequently utilize dynamic canopy sensing via thermal or multispectral imagery [18,19]. In areas lacking detailed soil maps or high-resolution terrain models, delineation workflows may depend on sparse soil sampling or freely available satellite data such as Sentinel-2 [20]. These examples demonstrate that fields with similar theoretical sources of variability may nevertheless require different sensing and computational strategies due to economic, agronomic, or logistical constraints.

While the diversity of available data provides a rich basis for characterizing within-field variability, its practical utility ultimately depends on the analytical methods used to extract, integrate, and translate these measurements into MZs. Methodological approaches have evolved alongside advances in data acquisition techniques. Geostatistical methods remain widely used for interpolating spatial patterns [7], and unsupervised clustering algorithms, particularly k-means and fuzzy c-means (FCM), continue to dominate practical MZ delineation due to their independence from costly labelled training data and their ability to capture the continuous spatial variation inherent in soil and crop processes [21]. Dimensionality reduction and multivariate data fusion methods facilitate the integration of heterogeneous datasets [22], while more recent studies [14,23] have explored machine learning approaches for MZ delineation or for predicting spatial patterns of yield potential and crop response. However, the operational adoption of machine learning in MZ delineation remains limited. Main barriers include the lack of labeled data, poor model transferability across different seasons and environments, and ongoing challenges in explaining and validating zone boundaries under real field conditions [24].

Despite substantial technological progress, the review literature on site-specific management remains fragmented. Existing syntheses typically emphasize only selected aspects of MZ delineation, leaving limited guidance on how field-scale workflows are operationally assembled and validated across diverse agronomic settings. A more explicit positioning of prior reviews and the resulting research gap is presented in Section 2.

The objective of this paper is to provide a systematic synthesis of the data sources, computational methods, and validation practices used in field-scale MZ delineation in precision agriculture. Based on a systematic analysis of 137 peer-reviewed studies published between 2000 and 2025, this work aims to address three key questions:

- What data sources and computational methods define current and emerging workflows?
- Where and under what conditions has MZ delineation been applied?
- How is the quality of MZs evaluated in practice?

By addressing these questions, the paper provides a coherent synthesis of operational practices while identifying methodological gaps and emerging trends across diverse agronomic contexts.

The remainder of this paper is structured as follows. A structured analysis of existing review literature and the resulting research gap is presented in Section 2. Section 3 describes the review protocol. Section 4 synthesizes the 137 studies, detailing their application contexts, the data and methods used for delineation, and the reported validation strategies. Section 5 interprets these findings and critically discusses the associated challenges, gaps, and emerging opportunities, along with future research directions. Finally, Section 6 concludes with a forward-looking perspective of the study’s implications.

## 2. Related Reviews and Research Gap

To clarify the specific gap this study addresses, existing reviews on MZ delineation were examined in terms of their primary scope and perspective. These studies synthesize specific aspects of the problem but do not provide an integrated perspective on how delineation pipelines are constructed in practice. They can be broadly categorized into three main groups:

- Management objective-oriented: Reviews focusing on specific decision goals, such as nutrient management [25,26] or irrigation management [27,28].
- Single sensing technology-oriented: Studies focusing on individual sensing modalities, such as geophysical sensing [29], remote sensing [28], and proximal sensing [30].
- Computational perspective-oriented: Reviews emphasizing algorithmic and methodological developments from data mining [31] or machine learning perspectives [32].

While these studies provide in-depth insights within their respective domains, they rarely examine how diverse sensing modalities, data layers, and computational techniques are integrated into practical MZ delineation workflows. Moreover, existing syntheses are often fragmented across geographic regions and cropping systems, with many studies tailored to specific agroecological contexts such as North African drylands [33], Japanese rice systems [34], Indo-Gangetic rice–wheat systems [35], or high-value perennial crops [30]. As a result, their findings are difficult to generalize across diverse production environments.

Taken together, these limitations reveal a lack of a systematic, data- and method-oriented synthesis of how field-scale MZ delineation is operationally implemented across studies, particularly regarding the integration of sensing, data structuring, delineation methods, and validation strategies. To address this gap, the present review analyzes 137 peer-reviewed studies to identify recurring workflow patterns, methodological combinations, and emerging trends across diverse agronomic contexts.

## 3. Review Methodology

This paper follows a systematic and transparent review and synthesis workflow designed to ensure methodological rigor and comparability across the included studies. The review was conducted in accordance with the PRISMA 2020 statement for systematic reviews [36], and the completed PRISMA 2020 checklist is provided in the Supplementary Materials, with adaptations appropriate for methodological synthesis in computational and precision agriculture research. Given the interdisciplinary nature of the topic and the large number of initial papers, a prioritization stage was incorporated prior to manual screening. This step combined journal-quality filtering (Q1 journals according to Journal Citation Reports or Scopus CiteScore) with automated relevance scoring to prioritize methodologically relevant studies. The prioritization procedure was designed to improve feasibility and consistency and did not replace manual eligibility assessment. Recent work has shown that semi-automated, researcher-in-the-loop workflows can improve the scalability of systematic reviews while maintaining human verification of inclusion decisions [37]. The review methodology follows a structured three-stage workflow: (i) literature search, (ii) prioritization and screening, and (iii) data extraction and coding. Each stage is described in detail in the following subsections. This workflow was designed to support a consistent synthesis of how MZ delineation is implemented, evaluated, and reported across the literature.

### 3.1. Literature Search

**Figure 1:**
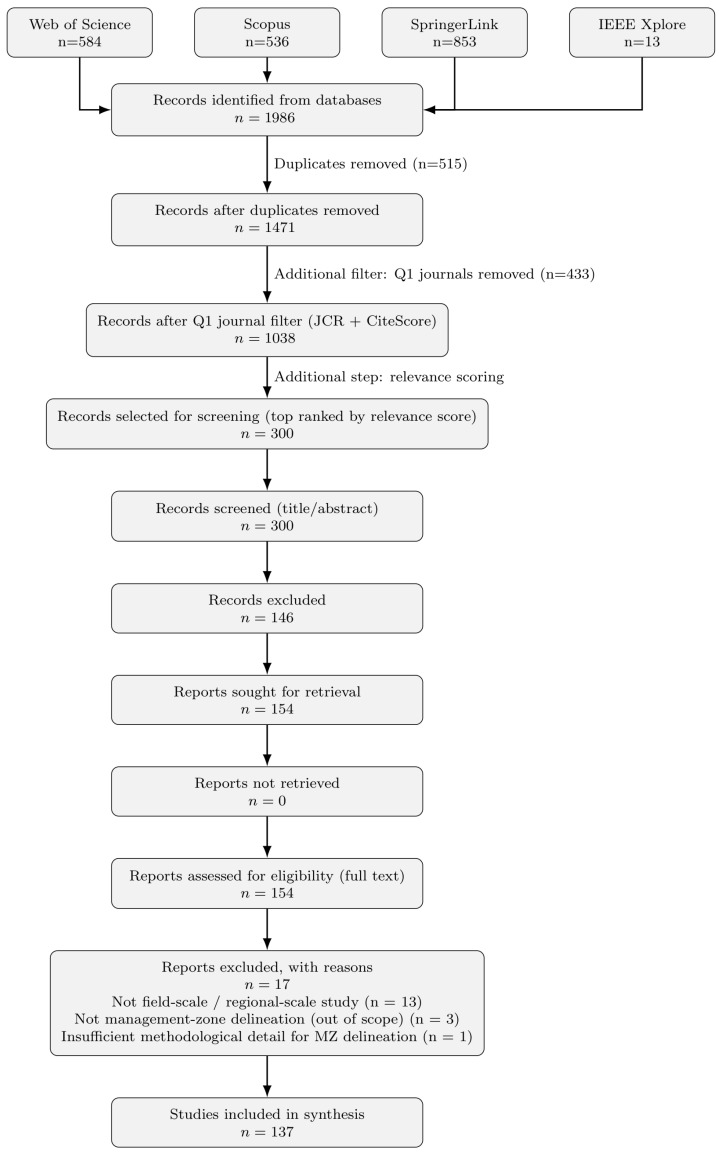
Study selection workflow for the management zone delineation review.

To ensure a level of the methodological transparency required for constructing our two-dimensional taxonomy, we restricted the scope to peer-reviewed journal articles published in English between 2000 and 2025 in Q1-ranked journals, as ranked by Journal Citation Reports (JCR) or Scopus CiteScore. While we acknowledge that this introduces potential selection bias by excluding practice-oriented studies from lower-tier venues, the criterion ensures that included papers provide sufficient technical detail to accurately classify MZ delineation workflows based on our taxonomy. This restriction prioritizes scientific maturation and experimental reproducibility over broad practical or on-farm utility. By focusing on top-tier publications, the review facilitates a synthesis of studies with well-documented experimental designs and clearly defined data-processing workflows, which are essential for identifying the field’s algorithmic evolution. We acknowledge that this may underrepresent some practical adaptations; however, it establishes a rigorous baseline for mapping the technological frontier of MZ delineation. The overall study selection workflow, following the PRISMA identification, screening, eligibility, and inclusion structure with an explicit prioritization step, is summarized in Figure 1.

#### Search Strategy

A structured literature search was conducted in October 2025 in the following databases: Web of Science Core Collection [38], Scopus [39], IEEE Xplore [40], and SpringerLink [41]. The search strategy combined three conceptual components: (i) management-zone terminology, (ii) methodological terms related to clustering, delineation, or segmentation, and (iii) precision-agriculture context.

- Management zone terminology (e.g., “management zone*”, “site-specific”, “variable rate”)
- Methodological terms (e.g., “cluster*”, “delineat*”, “segment*”)
- Precision agriculture context (e.g., “precision farming”, “smart agriculture”)

All searches covered 2000–2025 and were adapted to each database’s syntax. For instance, equivalent queries were applied in Scopus (TITLE-ABS-KEY) and SpringerLink (Keywords), with database-specific syntax adjustments. IEEE Xplore does not support complex multi-block Boolean expressions across abstract fields; therefore, a simplified but semantically consistent query was used.

In total, 1986 records were retrieved and imported into Zotero [42]. DOI-based deduplication yielded 1471 unique records. After restricting the results to Q1-ranked journals, 1038 papers with accessible abstracts were retained for relevance scoring.

### 3.2. Prioritization and Screening

As part of the prioritization stage, a keyword scoring scheme was applied to 1038 Q1 abstracts to identify the most methodologically relevant candidates for detailed screening. Keywords were grouped into six categories: (i) methods (e.g., “delineat*”, “cluster”; +5), (ii) data (e.g., “yield map”, “soil”; +4), (iii) application context (e.g., “precision agriculture”; +3), (iv) results/validation (e.g., “validation”, “R^2^”; +3), (v) general agricultural terms (e.g., “crop”; +1), and (vi) exclusion indicators (e.g., “survey”, “policy”; −5). Negative weights were assigned because such terms commonly occurred in studies that mentioned precision agriculture, yet did not implement spatial delineation. Therefore, the penalty helped deprioritize conceptually related but methodologically irrelevant papers.

Each category contributed its weight at most once per abstract, and a completeness bonus (+5) was assigned when method, data, and validation keywords were all present. Resulting scores ranged from −5 to 21 (mean ≈11.6). To ensure feasibility while maintaining systematic rigor, a predefined cap of 300 records was selected for manual screening. Accordingly, the highest-scoring 300 papers (score range: 18–21; mean ≈20.8) were advanced for detailed review. At the selection threshold, ties were resolved deterministically using: (i) a completeness indicator (presence of method, data, and validation signals), (ii) category-specific sub-scores (methods, data, results, application), and (iii) publication year. Remaining ties were resolved using a stable ordering by title to ensure full reproducibility of the final set.

Full keyword lists and scoring code are available in the public GitHub repository [43]. Relevance scores were used exclusively for prioritization and not as inclusion/exclusion criteria.

#### 3.2.1. Human-in-the-Loop AI-Assisted Screening

Following the prioritization stage, candidate studies were subjected to detailed screening and selection. Title and abstract screening of the top 300 papers was conducted manually by the primary author using predefined inclusion/exclusion criteria requiring: (i) a precision-agriculture context, (ii) empirical spatial data used to construct zones, and (iii) explicit methodological detail on delineation.

Following the initial manual screening conducted by the primary author, three large language models (LLMs) were incorporated as decision-support tools as a cross-validation step for title and abstract screening: Llama 3.1 8B [44], DeepSeek-R1 8B [45], and Gemini 2.5 Flash [46]. Each model received the title, authors, journal, year, and abstract together with the predefined screening criteria, and returned a binary INCLUDE/EXCLUDE decision, a confidence score, and a short textual rationale.

The AI-generated assessments were used only for cross-checking the initial human screening decisions. In cases where an LLM flagged a borderline study or disagreed with the primary author, the abstract was re-examined manually; all final inclusion and exclusion decisions strictly reflected the subject-matter expertise of the human reviewer.

This stage yielded 154 papers for full-text screening. To ensure the reliability of AI-assisted screening, the following validation procedure was implemented.

#### 3.2.2. Validation of AI-Assisted Screening

To assess the reliability of AI-assisted screening, all 300 records were first screened by the human reviewer based on the predefined inclusion and exclusion criteria. The decisions produced by each AI model were then compared against these human screening outcomes. Agreement was quantified using observed percentage agreement and Cohen’s kappa (κ), a standard measure of inter-rater reliability in systematic reviews [47]. Llama 3.1 showed substantial agreement with the human reviewer (81.0%, κ=0.610; n=300), while DeepSeek-R1 had moderate agreement (65.8%, κ=0.364; n=298, excluding two invalid outputs). Gemini 2.5 Flash processed a subset of records (37.7%) due to content-safety filtering but achieved substantial agreement on the evaluated subset (75.2%, κ=0.533; n=113). When both local models (Llama 3.1 and DeepSeek-R1) produced valid and matching decisions (the subset where AIs agreed), agreement with the human reviewer increased (84.6%, κ=0.694; n=201). These results indicate that AI outputs can provide a useful prioritization signal for identifying straightforward cases, while disagreement cases benefit from focused manual review.

Importantly, AI-assisted screening was used exclusively as a support mechanism rather than an automated decision process. All records were reviewed by a human reviewer, and all final inclusion and exclusion decisions were made by the reviewer based on predefined criteria. The full screening decision dataset, including human and AI model outputs, agreement status, and resolution notes, is available in the project repository (GitHub) as Supplementary Materials to ensure transparency and reproducibility [43].

#### 3.2.3. Full-Text Screening and Final Inclusion

Full-text versions were obtained for all 154 papers. Each article was assessed for: (i) sufficient methodological detail linking data sources, computational steps, and the resulting management zones; (ii) relevance to field-scale agriculture applications; and (iii) the presence of an identifiable validation or evaluation approach. Seventeen papers were excluded at this stage, primarily because they addressed regional or large-area zoning rather than field-scale MZ delineation or lacked sufficient methodological detail. This resulted in a final set of 137 included papers. Detailed exclusion reasons are documented in Appendix A, Table A1.

### 3.3. Data Extraction and Coding

For each included study, we extracted variables representing five major dimensions of MZ delineation: (i) agronomic and regional context (country, crop, field size, management focus); (ii) data used directly for delineation together with their associated sensing modalities; (iii) auxiliary data that supported preprocessing or environmental interpretation but were not inputs to the delineation algorithm; (iv) computational and analytical methods, including specific algorithms and the number of delineated zones; and (v) validation strategies and independent data used to evaluate zone quality, along with publication metadata for temporal trend analysis.

Due to the large number of papers, data extraction was conducted using a semi-automatic, human-in-the-loop workflow. Machine-generated draft summaries were produced using Google’s NotebookLM [48], which ingested the full text (PDF format) and generated structured summaries according to the predefined extraction fields. During an initial pilot phase (17 top-ranked papers), NotebookLM outputs were audited field-by-field against manually extracted entries to evaluate their usability as draft extractions and to identify common error modes (e.g., omissions vs. phrasing differences). The audit indicated that AI-generated summaries were generally complete and, in many cases, more comprehensive in capturing descriptive methodological details. Based on this evaluation, AI assistance was used to generate preliminary entries, while the primary author verified, corrected, and finalized every field using the source’s full text.

For transparency and reproducibility, the extraction prompt and template are included in Appendix B, and the associated code for the processing pipeline and extracted dataset are publicly available on the project’s GitHub repository and on Zenodo, respectively, [43,49].

#### Taxonomy Application and Consistency

The central analytical framework of this review is a two-dimensional taxonomy (data structuring strategies and zoning mechanisms), which is formally introduced in Section 4.4. This taxonomy was applied to each included study using predefined category definitions. Although the primary coding was conducted in a single pass, ambiguous or borderline cases were systematically revisited and re-evaluated in light of these definitions and the full methodological context of each study.

In addition to the primary two-dimensional taxonomy, auxiliary categorizations (e.g., data sources, validation approaches, sensing technologies, and management objectives) were defined using explicit category definitions and applied using the same definition-driven classification framework. Ambiguous cases within these categorizations were similarly revisited to maintain consistency.

Overall, the classification process emphasized explicit decision criteria and iterative verification of uncertain assignments. This approach reduces subjectivity by using predefined definitions and incorporating repeated evaluation of challenging cases, consistent with standard practices in qualitative systematic reviews. Together, these measures enhance reproducibility and transparency while ensuring feasibility at scale.

## 4. Systematic Synthesis of MZ Delineation

This section synthesizes the reviewed studies through a structured analysis to identify not only which methods and data sources have been used for MZ delineation, but also the underlying patterns, constraints, and drivers that shape these choices across different agronomic contexts. We begin with an overview of publication distribution in Section 4.1 to contextualize the reviewed literature with respect to temporal growth, geographic distribution, and agronomic diversity. Subsequent sections examine the role of data sources (Section 4.2) and sensing technologies (Section 4.3) as foundational inputs, followed by an analysis of computational methods and workflow structures (Section 4.4). We then investigate how these components are adapted to specific agronomic contexts (Section 4.5), and how their outcomes are evaluated in practice (Section 4.6).

Together, these analyses provide a connected view of how MZ delineation is operationally implemented, enabling identification of recurring workflow patterns, systemic limitations, and emerging opportunities for more robust and transferable delineation strategies.

### 4.1. General Characteristics of the Reviewed Papers

The final set of papers includes 137 peer-reviewed field-scale MZ delineation papers published between 2000 and 2025. The complete list of all reviewed papers, including their DOIs and extracted study-level characteristics, is provided as a searchable web-based supplementary database [49], accessible through an interactive interface at https://roghiheidari.github.io/mz-delineation-review-code/ (accessed on 10 May 2026) [43].

**Figure 2:**
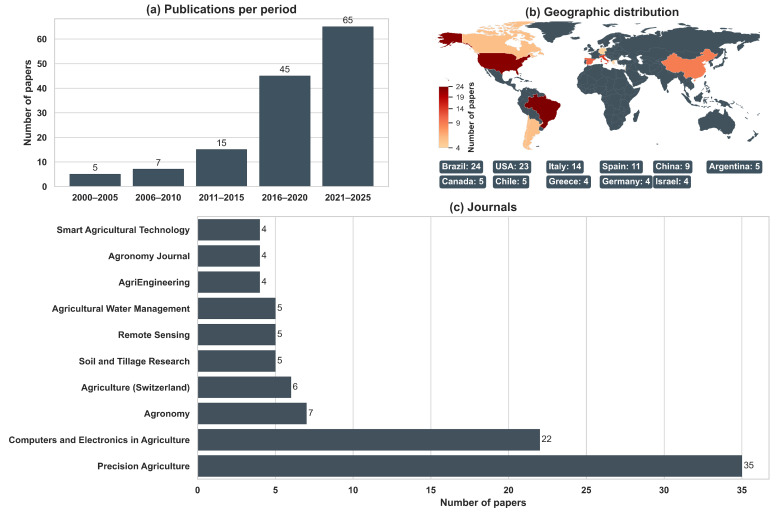
Overview of the reviewed papers. (**a**) Number of papers over the publication periods. (**b**) Distribution of papers across the most frequently represented countries. (**c**) Distribution of papers across the most frequently represented journals. Bar annotations indicate paper counts.

A temporal analysis (Figure 2a) shows a marked increase in publication activity, with rapid growth from 2016 onward. This acceleration coincides with the widespread availability of high-resolution satellite imagery (e.g., Sentinel-2), advances in proximal sensing technologies, and increased accessibility of machine learning tools. Beyond technological availability, this trend may also reflect a broader shift in precision agriculture from primarily experimental studies toward more operational applications.

Geographically, the literature is dominated by Brazil, the United States, Italy, Spain, and China, which together contribute more than half of all included papers (Figure 2b). These regions represent diverse production systems and crops, while a smaller but notable set of contributions originates from arid and semi-arid regions (e.g., parts of the U.S. [50], and Iran [51]), where irrigation and salinity management are central agronomic challenges.

The included papers span 26 different journals, with the majority appearing in *Precision Agriculture* and *Computers and Electronics in Agriculture* (Figure 2c). This distribution reflects the interdisciplinary nature of MZ research, which spans agronomy, soil science, remote sensing, and computational agriculture.

**Figure 3:**
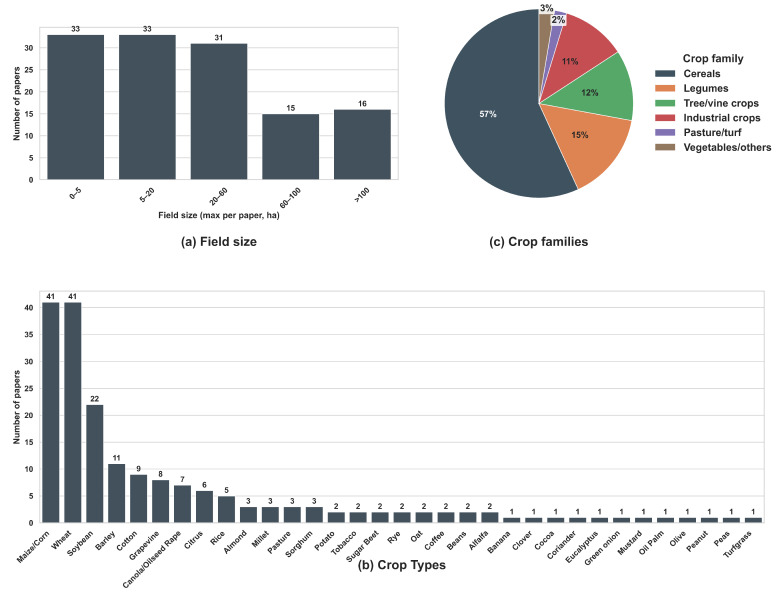
Field scales, crop types, and families in the reviewed papers. (**a**) Distribution of papers by maximum field size (ha) reported per paper. (**b**) Frequency of crop types investigated. (**c**) Proportional distribution of crop families across all papers. Numbers above bars and within pie segments indicate counts and percentages, respectively.

Following these general characteristics, the corpus also exhibits substantial diversity in field size and crop systems (Figure 3).

Field sizes range from smallholder plots to large commercial operations (Figure 3a). Approximately one quarter of the papers (33) were conducted in fields of less than 5 ha, demonstrating the applicability of MZ delineation even in small fields [18,52]. An equal number of papers (33) examined fields of 5–20 ha, including examples from Europe, the United States, and Brazil [53,54], though similar field sizes were reported in many other countries as well. A further 31 papers focused on fields between 20 and 60 ha, representing medium-scale commercial operations [55,56]. Larger commercial field sizes were also included in the reviewed studies, with 15 papers focusing on fields of 60–100 ha [57,58], and 16 papers considering fields exceeding 100 ha [59,60]. Overall, this distribution indicates that MZ delineation has been applied across a broad range of field scales, without a strong concentration in any single size category.

Crop types were highly diverse, but unevenly distributed (Figure 3b). Maize/corn and wheat (e.g., [61]) dominate the literature, with 41 papers each, reflecting their global importance and extensive adoption of precision agriculture. Soybean (22 papers), barley (11), and cotton (9) form the next tier of representation, followed by a wide range of specialty or region-specific crops, including grapevine (8). Several other crops (e.g., oat or banana) appear only once or twice. This low representation may reflect either the lower prevalence of these crops in the studied regions or limited research attention in the MZ literature.

At the crop family level (Figure 3c), cereals account for 57% of all papers, followed by legumes (15%), tree and vine crops (12%), and industrial crops (e.g., cotton, sugar beet) (11%), while pasture/turf systems and other crops together represent a small fraction of the dataset. This distribution indicates that MZ delineation research remains predominantly focused on grain production systems, with comparatively fewer studies in perennial and specialty crops.

Together, these characteristics provide context for the subsequent analysis of sensing modalities, data sources, and delineation methods. They show that MZ research has expanded substantially over time and has been applied across diverse geographic regions, field scales, and crop systems, although certain domains (e.g., specific crop types) remain less represented. These patterns form the basis for a more detailed examination of how delineation workflows are constructed and applied in practice.

### 4.2. Applied Data Sources for Management Zone Delineation

**Figure 4:**
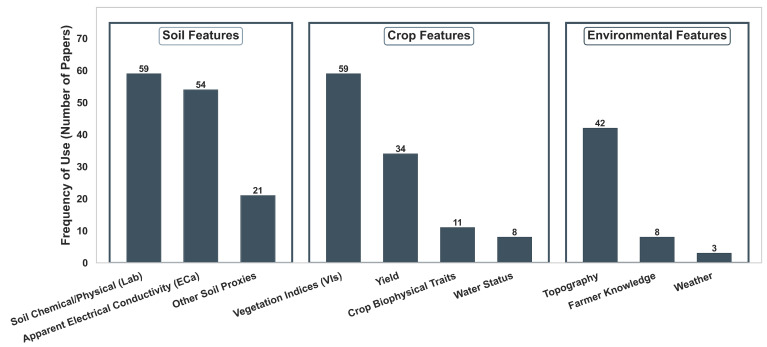
Frequency of data usage across reviewed papers, grouped into soil features, crop features, and environmental factors. Counts reflect the number of papers in which each data type was used as an input for MZ delineation.

The delineation of MZs relies heavily on the selection of input variables that accurately represent yield-limiting factors [25]. Across the reviewed papers, data sources are categorized into three primary groups of features (Figure 4): soil, crop, and environment. Throughout this section, features refer to the variables directly used for zone delineation, including both raw sensor or sampling measurements and variables derived through data processing (e.g., Normalized Difference Vegetation Index (NDVI)). Auxiliary information that supports preprocessing or data selection (e.g., using weather conditions to choose suitable satellite images) is not considered a direct input for MZ delineation. As illustrated in Figure 4, soil-related features are the most frequently utilized data source (cumulative frequency = 134), followed by crop features (112) and environmental features (53). This pattern is consistent with the common use of static and temporally stable indicators (e.g., soil texture, electrical conductivity, topography), complemented by dynamic vegetation and stress indicators captured throughout the growing season.

#### 4.2.1. Soil Features

Soil properties are the most common input data for MZ delineation due to their temporal stability, which is essential for defining long-term management strategies. Laboratory measurements of chemical and physical properties (e.g., nutrient concentrations, pH, texture) are widely used and typically collected via grid or other systematic sampling strategies (often ranging from dense 25–50 m grids to coarser resolutions of 1 sample per hectare, depending on field size and resources) [62,63]. For instance, authors in [64] utilized spatial variability of soil properties to define potential MZs. Similarly, authors in [65] delineated MZs for green onion production in Egypt based on site-specific nutrient status derived from soil analysis. Although accurate, the cost of dense soil sampling often leads researchers to combine it with other data, such as sensors.

A particularly common proxy for soil variability is apparent soil electrical conductivity ($EC_{a}$), which reflects the bulk electrical response of the soil measured in situ using proximal sensors (e.g., Veris, EM38). Unlike discrete grid sampling, these sensors typically collect high-density continuous data along parallel transects (e.g., 10 to 30 m apart) [66]. $EC_{a}$ integrates several influential soil features, including texture, moisture content, salinity, and clay mineralogy [67,68,69,70], and has been used widely in MZ delineation. For instance, authors in [71] demonstrated the efficacy of $EC_{a}$ for delineating productivity zones in claypan soils. In Brazil, $EC_{a}$ is utilized alongside 50 × 50 m grid sampling to delineate zones for grain and cocoa production, respectively, [63,72], confirming its utility across different cropping systems. In addition, $EC_{a}$ is frequently fused with other data. For example, $EC_{a}$ (at 2–3 m intervals) is combined with other data, such as soil features (laboratory measured), to improve nutrient management efficiency [66].

In addition to laboratory-measured soil properties and $EC_{a}$, a subset of studies incorporated alternative soil features (see “other soil proxies” in Figure 4) that capture soil variability through proximal sensing, remote sensing proxies, or legacy information. These include non-$EC_{a}$ proximal measurements such as Vis–NIR spectroscopy for predicting multiple soil properties [22,73,74,75], soil depth to restrictive layers [58,76,77], and soil moisture [78]. In addition, remotely sensed soil proxies derived from bare-soil imagery, such as soil brightness or color, were used to characterize soil organic matter (SOM) and texture patterns for subsequent MZ delineation [23,58,79,80], as well as modelled soil salinity from satellite time series [81]. Finally, legacy soil maps and databases (e.g., soil survey maps and soil series classifications) were directly incorporated as spatial inputs for MZ delineation [82,83,84,85].

Overall, the dominance of soil features, both laboratory and sensor-derived, highlights their central role in MZ delineation, especially in nutrient and fertility management applications.

#### 4.2.2. Crop Features

Crop features capture the direct integrated response to crop-limiting factors, such as soil, weather, and management conditions. For synthesis purposes, we grouped crop-related inputs into four categories: vegetation indices (VIs), yield data, crop biophysical traits, and crop water status indicators.

VIs are the most frequently used crop features (59 papers), reflecting their capacity to provide spatially continuous indicators of canopy condition and vigor [86]. They are derived from multispectral or hyperspectral imagery acquired through four principal platforms that differ in spatial resolution, temporal frequency, operational flexibility, and cost: satellite, UAV, aircraft, and proximal or ground-based sensing [87,88]. Regardless of the acquisition technology, VIs serve as integrative indicators that link spatial crop performance with underlying field variability [87,89].

Yield data are often considered the cumulative indicator of production constraints, and they are the second-most-used input for MZ delineation in this group. Yield maps are commonly utilized as an independent validation dataset (e.g., [3,58]); however, several papers also incorporated yield data directly as an input layer for MZ delineation (e.g., [90,91]). This dual role reflects the central importance of yield as both a performance benchmark and a spatial predictor.

Crop biophysical features, such as tree height or canopy nitrogen (N) content, are used directly in emerging MZ delineation frameworks [16,65]. These features are typically derived from proximal or remote sensing systems, including LiDAR [18], UAV-based photogrammetry [16], or airborne hyperspectral sensors [92], enabling the spatially continuous estimation of structural and compositional plant traits such as canopy height, leaf area, and biomass. They are particularly relevant in perennial cropping systems or high-resolution phenotyping contexts [18].

Crop water status metrics, such as the Crop Water Stress Index (CWSI), are used in eight papers to delineate irrigation zones based on thermal indices (e.g., [93,94]).

#### 4.2.3. Environmental Factors

Environmental indicators were used in 53 papers and included topography and terrain features (42 papers) (e.g., [82,95]), farmer knowledge (eight papers) (e.g., [56,96]), and three weather-driven features [97,98,99]. Topographic features such as elevation, slope, curvature, and the topographic position index (TPI) are widely used and associated with processes including water redistribution, erosion patterns, and soil formation [11].

Farmer and expert knowledge contribute to MZ delineation either through direct manual mapping based on field experience or by embedding qualitative expertise into computational frameworks (e.g., fuzzy inference systems), enabling automated zones to better reflect practical field variability and yield-limiting factors [56,79,83,100].

Weather features are rarely used as direct inputs for MZ delineation and are typically limited to specific applications, such as pest management [98] and hydrologic classification [99].

#### 4.2.4. Evolution and Integration Patterns of Data Sources

Consistent with previous comprehensive reviews of the field, the reviewed literature indicates that no single data source or group of sources consistently yields superior MZ delineation outcomes across all agroecological contexts [25]. Instead, the reviewed literature indicates a shift toward data fusion workflows that combine static soil information with dynamic crop-based sensing. Such integrated approaches capture both structural heterogeneity and seasonal variability, and are frequently adopted in the reviewed papers.

**Figure 5:**
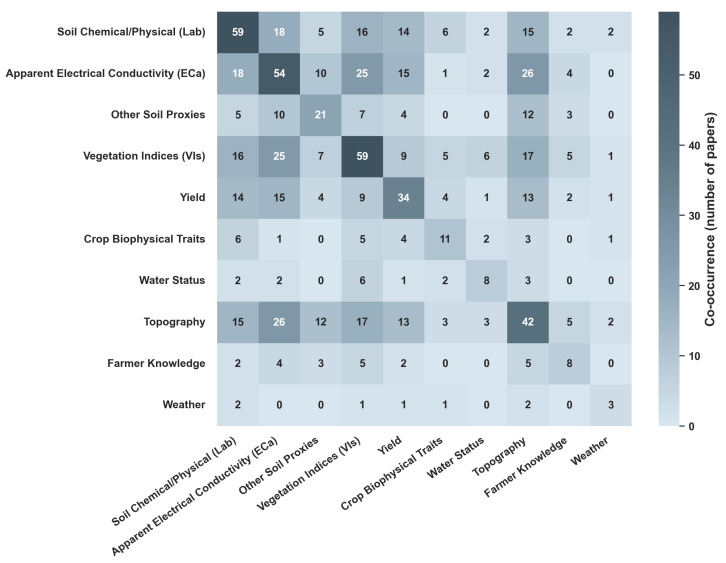
Co-occurrence frequency of data sources used for MZ delineation across the reviewed studies. Diagonal values indicate individual usage frequency, while off-diagonal values represent the number of papers in which two data sources were used jointly.

Figure 5 further quantifies this transition toward multisource integration. High co-occurrence frequencies among apparent electrical conductivity ($EC_{a}$), VIs, yield data, soil physicochemical, and topographic features indicate that these sources are among the most commonly combined in contemporary MZ workflows. In contrast, weather variables, water status indicators, and farmer knowledge appear less frequently and are rarely integrated simultaneously with multiple other data streams. This pattern suggests that spatially stable structural proxies and remotely sensed indicators are more commonly prioritized, while dynamic environmental and experiential knowledge sources are less frequently incorporated.

Early research (approximately 2000–2010) typically relied on yield monitor data, soil laboratory measurements, and proximal sensing, particularly apparent electrical conductivity ($EC_{a}$), as primary proxies for spatial variability, consistent with their relationships to soil texture and historical productivity [71,101]. Beginning in the mid-2010s, the integration of stable topographic features (e.g., elevation, slope) with multispectral imagery became increasingly common, combining terrain-driven structure with crop response indicators [59,66].

In the more recent period (post-2018), the availability of open-access satellite data (e.g., Sentinel-2) and commercial constellations (e.g., PlanetScope) has facilitated broader use of multi-temporal VIs and phenological metrics [55,81,102]. Contemporary studies increasingly employ data fusion frameworks that integrate optical, thermal, and radar data with machine-learning approaches, thereby partially reducing reliance on intensive soil sampling and facilitating season-specific zoning that accounts for temporal variability [23,60,103].

### 4.3. Applied Sensing Technologies for Management Zone Delineation

The delineation of MZs relies on data capable of resolving within-field variability at agronomically relevant scales. Based on the reviewed papers, a range of sensing technologies is used to generate such data for zoning algorithms. Excluding sensors used solely for validation, sensing approaches are categorized according to deployment platform and operational context into four groups: proximal soil sensing, proximal crop sensing, airborne and spaceborne remote sensing, and yield mapping (Table 1).

#### 4.3.1. Proximal Soil Sensing

Proximal soil sensing (PSS) systems are ground-based instruments, operated manually or mounted on vehicles in proximity to the soil surface (typically within 2 m), designed to rapidly and cost-effectively acquire information on within-field soil variability [23,104]. Measurements can be acquired either at discrete stationary sampling points or continuously in motion (on-the-go sensing), depending on the sensor type and deployment configuration. In the reviewed literature, $EC_{a}$ sensors are predominantly deployed as mobile, vehicle-towed platforms to achieve high-density spatial coverage [68,71,105]. In contrast, optical and mechanical sensors are more commonly applied at discrete sampling locations, although recent developments have enabled their integration into on-the-go platforms [22,78].

By enabling dense spatial sampling without extensive laboratory analysis, PSS has become a central tool for mapping soil heterogeneity in MZ delineation. Based on their underlying physical measurement principles, proximal soil sensors are generally classified into three categories: electrical/electromagnetic, optical/radiometric, and mechanical [104].

– **Electrical and Electromagnetic Sensors:** The most extensively utilized PSS technology in the reviewed literature is the measurement of $EC_{a}$ [63,71,101]. These sensors operate through two main interaction modes: non-contact electromagnetic induction (EMI) and galvanic contact resistivity. EMI systems (e.g., Geonics EM38 [71], DUALEM [106]) generate an electromagnetic field that induces secondary currents within the soil, allowing conductivity to be measured without direct contact. Dual-geometry EMI configurations employ multiple transmitter–receiver coil orientations to capture conductivity responses at different depths [106]. In contrast, galvanic contact systems (e.g., Veris 3100 [66], automatic resistivity profiling systems [107]) inject electrical current into the soil through electrodes and measure the resulting voltage response to estimate resistivity. $EC_{a}$ measurements serve as robust proxies for spatially variable yet relatively stable soil physical properties, primarily soil texture (clay content), moisture, and salinity [68,69]. Through these relationships, $EC_{a}$ is frequently associated with chemical fertility indicators such as Cation Exchange Capacity (CEC), SOM, and pH, particularly in saline or claypan soils [23,66].
– **Optical and Radiometric Sensors:** This category includes passive gamma-ray spectrometry and active optical reflectance sensors. Gamma-ray spectrometers (e.g., The Mole, Medusa MS1200, SoilOptix) characterize soil mineralogy, texture, and total organic carbon by passively measuring naturally emitted gamma radiation from the soil surface and relating radiometric signal intensity to soil compositional attributes through calibration with laboratory samples [23,104]. Furthermore, online visible- and near-infrared spectroscopy platforms (e.g., Tec5) estimate multiple soil fertility parameters (e.g., pH, organic carbon, phosphorus, potassium (K), and magnesium) by analyzing wavelength-dependent reflectance signatures associated with molecular absorption features, enabling simultaneous prediction of chemical properties during field traversals [22,75].
– **Mechanical Sensors:** Mechanical sensors quantify soil mechanical resistance to identify compaction layers that may restrict root development. Devices such as instrumented cone penetrometers (e.g., Falker PenetroLOG, Toro Precision Sense) map Soil Penetration Resistance (SPR) by inserting a standardized probe into the soil at a controlled rate and recording the force required to advance through the profile, thereby providing depth-resolved resistance measurements [78,108]. These spatially distributed resistance profiles are used to detect physical limiting layers and support MZ delineation [78,109].

#### 4.3.2. Proximal Crop Sensing

Proximal crop sensing involves the use of ground-based active optical sensors to assess crop vigor and nutritional status in real-time or for high-resolution mapping. Unlike passive sensors, these devices emit their own modulated light source, making them independent of solar illumination conditions.

The most frequently cited sensor in this category is the GreenSeeker (Trimble Inc., Westminster, CO, USA), which measures canopy reflectance in the red and near-infrared (NIR) bands to calculate NDVI [86,110]. Other active sensors, such as the Yara N-Sensor ALS, are used to derive indices utilizing the Red Edge band (e.g., NDRE) to overcome saturation issues associated with NDVI at high biomass stages [111]. These sensors are often mounted on tractors or high-clearance vehicles to capture crop phenotypic data that informs dynamic, in-season MZ delineation strategies [111,112].

**Table 1:** Top sensors used directly for MZ delineation in the reviewed papers.

Sensor Category	Sensor Sub-Category	Sensed Variable(s)	Brand & Model	Papers
Proximal Soil Sensing	Electrical & Electromagnetic (EMI)	Apparent Electrical Conductivity ($EC_{a}$)	Geonics EM38, EM38-MK2, EM38-DD; GSSI Profiler EMP-400; Geophex GEM-2	[3,68,69,71,104,113,114,115]
	Electrical & Electromagnetic (Galvanic Contact)	Apparent Electrical Conductivity ($EC_{a}$), Electrical Resistivity (ER)	Veris 3100, 3150, MSP3; Geocarta ARP; LandMapper ERM-02	[2,23,58,63,66,71,72,82,101,107,111,116]
	Electrical & Electromagnetic (EMI—Dual Geometry)	Apparent Electrical Conductivity ($EC_{a}$)	DUALEM (1S, 21S, 2)	[18,105,106,117]
	Optical & Radiometric (Vis-NIR Spectroscopy)	Soil Spectra (predicting SOM, pH, Moisture, P, K)	Tec5 CompactSpec	[22,73,74,75]
	Optical & Radiometric (Gamma-Ray)	Natural Gamma Radiation (K, U, Th), Soil Texture	Medusa MS1200, The Mole, SoilOptix	[23,104]
	Mechanical	Soil Penetration Resistance (SPR), Compaction	Falker PenetroLOG; Toro Precision Sense 6000	[78,109]
Proximal Crop Sensing	Optical (Active)	NDVI, NDRE, Canopy Reflectance	GreenSeeker (Trimble), Crop Circle (Holland Scientific), N-Sensor ALS (Yara)	[77,86,110,111]
	Thermal	Canopy Temperature ($T_{c}$)	Infrared Thermometers (IRTs)	[112]
Remote Sensing (Satellite)	Optical (Multispectral)	Reflectance (VNIR, SWIR), VIs (e.g., NDVI)	Sentinel-2 (MSI)	[20,81,88,103,118,119,120]
	Optical (Multispectral)	Reflectance (VNIR, SWIR), VIs (e.g., NDVI)	Landsat 5, 7, 8	[50,121,122]
	Optical (High Resolution)	Reflectance (VNIR), VIs	PlanetScope (Planet Labs), RapidEye, WorldView-2	[59,69,83,89,102,123,124]
	Thermal	Evapotranspiration (ET), Land Surface Temperature (LST)	MODIS, Landsat (TIRS)	[55]
	Radar (SAR)	Backscatter coefficients (VV, VH)	Sentinel-1	[60,113]
Remote Sensing (UAV)	Optical (Multispectral)	Reflectance (VNIR, Red Edge), VIs	MicaSense (RedEdge, RedEdge-MX, Altum); Parrot Sequoia; DJI (P4 Multispectral, Mavic 3M); Mapir Survey 3	[3,16,19,52,69,94,98,113,125,126,127]
	Thermal	Canopy Temperature ($T_{c}$), CWSI	FLIR (e.g., SC655, SC2000, Duo Pro R, Tau 2), Zenmuse XTS	[87,94,112]
Remote Sensing (Aircraft)	Optical & Thermal	Reflectance, Canopy Temperature, CWSI	FLIR A65, SpecTerra DMSC, HyMap, AVNIR	[92,93,128,129,130]
Yield Mapping	Mechanical/Optical	Grain/Crop Yield, Moisture	Yield Monitors (Ag Leader, John Deere, Case, CLAAS)	[2,23,71,84,90,91,96,97,131,132,133,134,135]

#### 4.3.3. Airborne and Spaceborne Remote Sensing Platforms

This group includes satellites, manned aircraft, and UAVs. These platforms enable non-invasive monitoring of crop and soil variability across multiple spatial and temporal scales, making them particularly well-suited for large-area and multi-temporal MZ delineation. Crucially, the multi-temporal acquisition capabilities of these platforms facilitate time-series analysis, allowing for crop phenology tracking throughout the season, assessing the temporal stability of MZs, and distinguishing short-term environmental stress from structurally persistent yield-limiting patterns [81,120]. While several proximal crop sensing systems also rely on non-contact radiometric principles, they are treated separately due to their ground-based deployment and field-scale operation.

Satellite imagery is extensively used (Table 1) due to the availability of free, high-temporal-resolution data. Optical satellites operate as passive sensors, measuring solar radiation reflected from the Earth’s surface across multiple spectral wavelengths to assess vegetation condition [81]. A primary example is the Sentinel-2 constellation, which provides 13 spectral bands, including visible, red-edge, near-infrared, and shortwave infrared, at spatial resolutions ranging from 10 to 60 m, with a 3–5-day revisit time under the two-satellite configuration [81,127]. This configuration enables continuous derivation of VIs (e.g., NDVI) to proxy crop vigour and monitor intra-seasonal variability [103]. While Sentinel-2 lacks a thermal band, Landsat data are commonly employed to capture canopy temperature and evapotranspiration (ET) patterns [55]. To overcome the spatiotemporal limitations of public satellites (10–30 m), high-resolution commercial constellations such as PlanetScope (3 m resolution, daily revisit) are increasingly utilized to detect temporary yield-limiting factors and delineate MZs, particularly in smallholder farming systems [69,102].

Because optical sensors are constrained by cloud cover and illumination conditions, recent studies increasingly integrate Synthetic Aperture Radar (SAR) data (e.g., Sentinel-1), which enables acquisition independent of atmospheric conditions. Unlike optical systems, SAR emits its own microwave energy (e.g., C-band) and records the backscattered signal, enabling data acquisition independent of cloud cover and solar illumination [60]. Sentinel-1 provides dual-polarization imagery at approximately 20 m spatial resolution, with an effective revisit interval of about 6–12 days depending on latitude and orbital configuration. Because microwave backscatter intensity is strongly influenced by surface roughness and dielectric properties, primarily governed by soil moisture content, SAR serves as a physically grounded proxy for mapping soil moisture variability independent of varying atmospheric conditions [60].

For higher spatial precision compared to satellites, manned aircraft equipped with multispectral and thermal sensors serve as an intermediate platform, effective for mapping variability in large orchard systems where row-level detail is required [93,128]. These airborne platforms have also proven effective in viticulture, where high-resolution vigor maps (NDVI) were successfully correlated with berry composition, acidity, and sanitary status (e.g., bunch rot incidence), facilitating selective harvesting strategies [129].

At the finest scale, UAVs function as low-altitude, flexible sensing platforms that can be equipped with interchangeable lightweight sensors (e.g., RGB, multispectral, and thermal infrared cameras), enabling ultra-high spatial resolution data acquisition (often at the centimeter level) [94,125]. This sensor–platform integration allows UAVs to provide highly detailed spatial information essential for separating pure canopy pixels from soil background and shadows in high-value row crops and orchards [19,125].

Beyond canopy segmentation, this high spatial resolution enables the classification of specific biotic constraints, such as discriminating between maize crops, weeds, and bare soil using visible-band indices like the Excess Green Index (EGI) [126]. Furthermore, UAVs offer spectral flexibility; for instance, the Red Edge band has been utilized to calculate the Normalized Difference Red Edge (NDRE) index to delineate MZs in clover-grass mixtures, overcoming saturation issues common with NDVI in dense canopies [52]. UAV-based thermal imagery is particularly valuable for calculating the crop water stress to delineate dynamic irrigation MZs [94].

Although UAVs can be deployed on demand and may operate below cloud cover in temperate regions [127], this flexibility is accompanied by higher operational costs, regulatory constraints, and limited spatial coverage compared to satellite-based observations. Sustaining high temporal frequency monitoring requires repeated field missions, flight planning, and data processing, which introduces logistical and economic constraints. In contrast to the automated revisit cycles of satellite constellations, UAV-based monitoring is therefore less scalable for continuous, large-area temporal assessment [69,94]. These operational considerations are reflected in the empirical adoption patterns reported in the reviewed literature.

Among studies that directly employed VIs for MZ delineation, satellite imagery was the most widely used acquisition platform, appearing in approximately 54% of the reviewed papers (e.g., [50,59,134]). UAV platforms were utilized in approximately 22% of studies, while proximal crop sensing systems and manned aircraft each accounted for around 12%. The observed distribution highlights how platform selection is primarily influenced by scalability and coverage requirements: satellite systems dominate large-area, multi-temporal applications; UAV and aircraft platforms are preferred when fine spatial detail is critical; and proximal sensing systems are adopted when real-time, canopy-level measurements are integrated within routine field operations.

**Figure 6:**
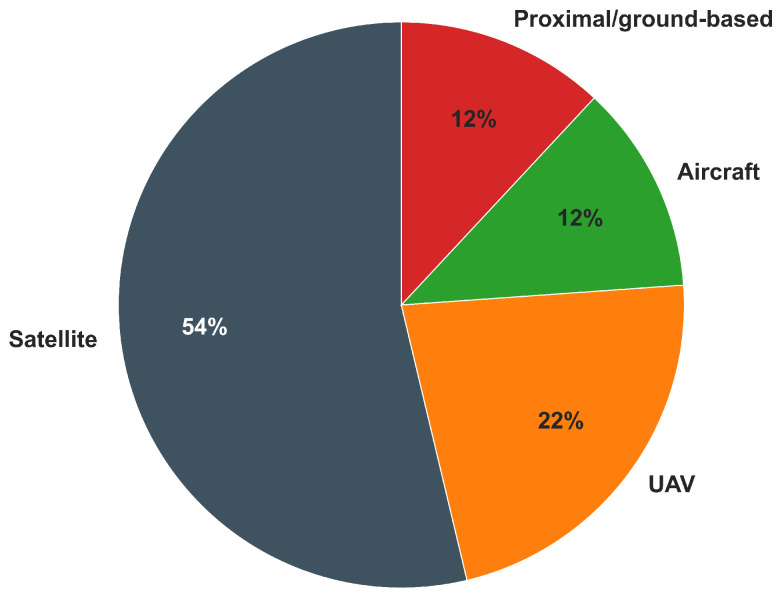
Relative frequency of acquisition platforms for VI-based MZ delineation in the reviewed literature.

Figure 6 summarizes the relative distribution of acquisition platforms used for VI-based MZ delineation in the reviewed literature.

#### 4.3.4. Yield Mapping

Yield mapping sensors mounted on combine harvesters provide the most direct measure of the integrated effect of soil and environmental factors on crop performance. Specifically, recording grain mass flow and moisture content and linking these measurements to GNSS (e.g., GPS) coordinates captures how spatial variability in soil texture, nutrient availability, topography, and seasonal weather conditions collectively influences final biomass accumulation and grain production [23,131]. Geo-referenced yield maps are therefore considered the standard reference for defining productivity zones [71,84].

To ensure reliability, multi-year yield data are often required to distinguish between temporally stable yield patterns (attributed to soil properties) and temporal variability mostly caused by weather [90,132]. However, raw yield data frequently contain systematic errors and outliers arising from mechanical and operational artifacts, variable harvester speed, partial or unknown header width during turns, and GNSS positioning inaccuracies and overlapped passes [82,132]. Consequently, robust post-processing, such as lag correction, spatial filtering, and outlier removal, is essential before using yield datasets for MZ delineation [132].

Yield stability maps derived from the temporal analysis of yield data are frequently fused with soil and topographic features to create robust MZs [23], or used to identify low-performing areas for precision conservation based on economic gross margins [131].

### 4.4. Algorithmic Workflows: Computational Methods for MZ Delineation

The reviewed literature reveals substantial methodological heterogeneity in MZ delineation approaches. Papers differ not only in the choice of algorithms, but also in how input data are structured and transformed before applying that algorithm. This diversity makes direct comparison of isolated algorithms insufficient for capturing the analytical architecture underlying MZ delineation.

**Figure 7:**
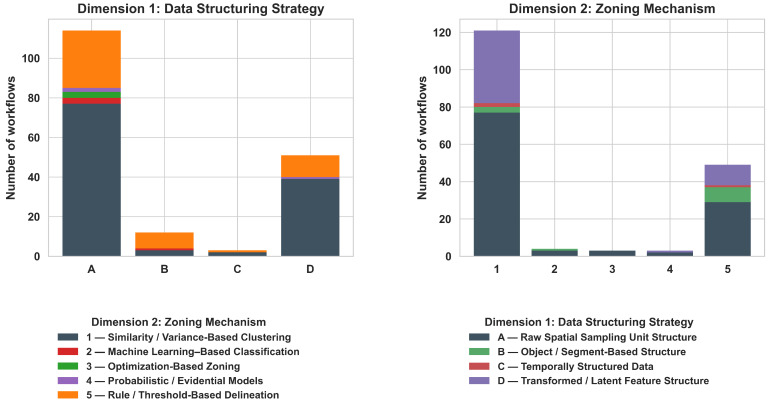
Two-dimensional taxonomy of MZ delineation workflows. The left panel shows the prevalence of zoning mechanisms (1–5) within each data structuring strategy (A–D); the right panel shows the reverse distribution. Within each stacked bar, colored segments represent the secondary dimension: in the left panel, colors correspond to zoning mechanisms (1–5), and in the right panel, colors correspond to data structuring strategies (A–D). Bars represent counts of independently classified workflows. Only algorithms that directly perform spatial partitioning are included.

To systematically compare the heterogeneous methodological landscape of MZ delineation, we summarize it as *workflows* rather than as isolated algorithms. Each workflow is encoded using a two-character alphanumeric code that captures (i) the *data structuring strategy* immediately before zoning (A–D) and (ii) the *zoning mechanism* that directly assigns discrete MZs (1–5). Importantly, only steps that perform spatial partitioning are classified; validation, evaluation, and post hoc analyses (e.g., ANOVA, Tukey tests, cross-validation, and yield comparisons) are excluded because they do not generate zone assignments. Furthermore, when a single paper includes multiple independent zoning workflows (e.g., [19,134]), each workflow is counted as a separate workflow. Figure 7 provides a compact overview of how workflows distribute across the two dimensions.

Across the reviewed literature, zoning remains dominated by raw spatial sampling units (Category A), while similarity/variance-based clustering (Class 1) constitutes the prevailing delineation mechanism. This dominance is not random but reflects several structural and practical constraints in precision agriculture. First, most sensing systems (e.g., soil sensors, satellite imagery, yield monitors) inherently produce raster- or point-based data, making pixel-level representations (Category A) the most direct and lossless form for analysis. Transitioning to object-based (B) or temporal (C) representations requires additional preprocessing, making them less commonly used. Second, clustering-based methods (Class 1) offer a computationally efficient and assumption-light framework for partitioning high-dimensional agronomic data without requiring labeled training data. This is particularly important in agricultural contexts, where ground-truth labels for optimal zoning are rarely available or costly to obtain. In contrast, more advanced approaches, including machine learning classification, optimization-based, and probabilistic methods (Classes 2–4), appear less frequently as direct zoning mechanisms because they introduce additional complexity and often require external inputs such as training data, constraints, or prior assumptions. As a result, these methods are more commonly integrated as supporting components, such as feature construction [23,62], constraint enforcement [136,137], or uncertainty modeling [83], rather than replacing clustering as the dominant final partitioning step. Overall, these patterns suggest that the application of clustering-based zoning is driven primarily by the alignment among data availability, computational simplicity, and operational feasibility in real-world agricultural systems. Methodological innovation in MZ delineation is therefore concentrated more in data preprocessing and feature engineering stages than in fundamentally new zoning paradigms.

#### 4.4.1. Dimension 1: Data Structuring Strategy

The first dimension (Categories A–D) characterizes the structural organization of input data before MZ delineation. This dimension captures how input data is organized, aggregated, or transformed before the application of any zoning algorithm, and is therefore independent of the mathematical MZ delineation mechanism employed.

(A) **Raw Spatial Sampling Unit Structure:** In Category A, zoning is performed directly on point observations, raster cells, or interpolated grid surfaces. Interpolation techniques (e.g., ordinary kriging, inverse distance weighting) are included within this category provided that the resulting surfaces are used in their original feature space without subsequent latent-space transformation or dimensionality reduction.

As illustrated in Figure 7, the raw spatial sampling unit structuring strategy (Category A) is the most widely used category in the reviewed literature. Many papers interpolate soil, terrain, vegetation, or yield data into continuous surfaces and apply zoning directly at the pixel level [3,6,90]. Importantly, the use of machine learning models does not alter the structuring classification when inputs remain spatially explicit grids (e.g., VIs, biomass estimates) without prior feature-space transformation [88,102].

In this category, the native spatial granularity of observations is preserved, and each pixel or sampling point is treated as an independent decision unit. This approach emphasizes local variability and fine-scale heterogeneity, often resulting in high-spatial-resolution MZ boundaries. While this representation preserves maximum spatial detail, it often results in fragmented zoning patterns, motivating the development of alternative structuring strategies, such as object-based aggregation (Category B).

(B) **Object-/Segment-Based Structure:** Category B comprises approaches in which zoning is performed on segmented spatial units rather than on individual pixels or point samples. Contiguous pixels are grouped into internally homogeneous objects prior to zone delineation, thereby introducing an intermediate spatial abstraction layer between raw measurements and final MZs. This structural segmentation reduces the salt-and-pepper fragmentation commonly observed in pixel-based approaches and enhances spatial coherence in delineated zones [92,138].

The segments are typically generated using Object-Based Image Analysis (OBIA) or multiresolution image segmentation algorithms. Fractal Net Evolution Approach (FNEA) and hierarchical multiscale segmentation methods are utilized to partition multispectral or high-resolution imagery into spectrally and geometrically homogeneous objects prior to zoning [124,139].

Beyond conventional satellite imagery, object-oriented segmentation has also been extended to hyperspectral data and time-series VIs, where multiresolution segmentation is used to maximize correlations with agronomic variables such as crop yield [92,138].

Conceptually, Category B redefines the fundamental decision unit of zoning from discrete pixels to spatially coherent entities. This shift enhances structural continuity, improves interpretability at the field-management scale, and often aligns more closely with operational agricultural boundaries. However, the effectiveness of segmentation depends strongly on parameter selection and image quality, which limits its robustness across diverse agricultural contexts and partially explains its lower adoption compared to pixel-based approaches.

(C) **Temporally Structured Data:** Category C includes approaches in which temporal sequences are explicitly preserved as part of the structural representation prior to zoning. In contrast to workflows that collapse multi-date imagery into static composites (e.g., [20,123,124]), which thereby reverting them to Category A, this category treats chronological dynamics as an intrinsic modeling dimension rather than a derived summary feature.

Under this strategy, spatial units are represented by temporal trajectories (e.g., VIs, ET, radar backscatter), and similarity is defined over their sequential evolution. Time-series clustering methods group spatial entities based on shared seasonal dynamics or phenological behavior [55]. In other implementations, high-frequency radar or optical signals are encoded as ordered vectors and either clustered directly or transformed using sequence-aware architectures (e.g., temporal autoencoders) to extract latent dynamic patterns prior to zoning [60]. Stability-based frameworks further leverage temporal variance metrics derived from historical satellite images to delineate MZs reflecting long-term consistency or variability in crop performance [118].

Category C extends the dimensionality of zoning from purely spatial heterogeneity to spatiotemporal behavior. The fundamental decision unit is no longer defined solely by its spatial attributes at a given time, but by its trajectory through time. Despite its ability to capture temporal dynamics, the increased data requirements and computational complexity have constrained its broader application in operational MZ delineation.

(D) **Transformed/Latent Feature Structure:** Category D comprises approaches in which input variables are projected into a transformed or reduced-dimensional feature space prior to zone delineation. Rather than operating directly on measured spatial features or temporal trajectories, these methods redefine the input data space through linear or non-linear embeddings before zoning.

Common transformations include principal component analysis (PCA) and spatial PCA [5,76,140], composite index construction and scaling frameworks [62,101], as well as non-linear encoders such as autoencoders and other latent embedding techniques [62,74]. When interpolated spatial grids or temporal sequences are subjected to such transformations before zoning, the workflow is categorized as D rather than A or C, as the zoning mechanism operates on derived latent variables rather than on original observations.

This structuring strategy is particularly suited to high-dimensional and multicollinear agricultural datasets, where redundancy among soil, terrain, crop, and remote sensing variables can hide meaningful structure [2,20,74,141]. By decomposing correlated predictors into orthogonal components or compressing them into non-linear latent representations, Category D emphasizes information abstraction over direct measurement representation to effectively manage multicollinearity and synthesize spatial variability [5,58]. Consequently, zoning outcomes reflect patterns expressed in transformed feature space rather than in the original spatial or temporal variables. While this abstraction enhances statistical efficiency and mitigates multicollinearity, it often reduces direct interpretability, as derived components or latent embeddings may not correspond to physically measurable agronomic variables. This introduces a trade-off between dimensionality reduction and explanatory transparency in MZ delineation [62,74,83].

**Synthesis of Data Structuring Strategies:** Across the reviewed literature, raw spatial sampling unit structuring (Category A) remains the foundational representation paradigm, particularly for soil, VIs, terrain, and yield datasets. However, a gradual methodological shift is evident toward transformed or latent feature representations (Category D), reflecting an increasing emphasis on dimensionality reduction, mitigating multicollinearity, and abstracting high-dimensional inputs. Object-based segmentation (Category B) provides an intermediate structural alternative that enhances spatial coherence and operational interpretability, while temporally structured representations (Category C) remain comparatively limited but signal growing recognition of spatiotemporal dynamics in zone delineation.

Overall, the evolution of Dimension 1 reflects a gradual transition from direct measurement-based representations toward more structured and abstract data formulations. However, this transition remains constrained by practical considerations, including data availability, computational cost, and interpretability. Specifically, while transformed representations (Category D) address multicollinearity and enable more compact feature spaces, they often reduce transparency and hinder agronomic interpretability. Similarly, object-based (B) and temporal (C) representations introduce additional modeling complexity and preprocessing requirements, which can limit their scalability and reproducibility across different fields and cropping systems. As a result, the continued dominance of raw spatial representations (Category A) highlights a fundamental trade-off in MZ delineation between methodological sophistication and operational feasibility. This trade-off remains a key factor shaping the methodological landscape of the field.

#### 4.4.2. Dimension 2: Zoning Mechanism

The second dimension of the taxonomy characterizes the computational logic used to partition spatial units into MZs. Whereas Dimension 1 defines how data are structurally represented and organized before delineation, Dimension 2 specifies how MZ boundaries are algorithmically determined within the defined feature space. Five principal classes of zoning mechanisms are identified: (1) Similarity/Variance-Based Clustering, (2) Machine Learning Classification, (3) Optimization-Based Methods, (4) Probabilistic/Evidential Methods, and (5) Rule-/Threshold-Based Methods.

**Class (1) Similarity/Variance-Based Clustering:** Unsupervised clustering constitutes the dominant zoning mechanism in the reviewed literature. These methods partition spatial units by minimizing within-zone variance while maximizing between-zones dissimilarity, thereby identifying statistically homogeneous regions without requiring labeled training data.

Fuzzy c-Means (FCM) is the most frequently reported algorithm [58,140]. Its soft-membership formulation is particularly suited to agronomic landscapes, where soil and crop properties vary continuously rather than exhibiting abrupt discontinuities. Hard-partitioning algorithms such as K-means and hierarchical clustering (e.g., Ward’s linkage) are also widely employed across both raw and transformed feature spaces [2,142]. The mechanisms in this class delineate MZs through intrinsic statistical similarity in feature space.

**Class (2) Machine Learning Classification:** Supervised learning algorithms, such as random forests, support vector machines, and neural networks, are utilized to classify homogeneous spatial units based on complex, non-linear relationships among features [88,112]. Unlike unsupervised clustering, these methods require labeled training data or predefined class definitions. In some workflows, supervised predictions directly define MZs [88,139]; more commonly, machine learning models function as feature construction or variable selection components prior to clustering-based delineation [23,62]. Class 2 therefore emphasizes predictive modeling across complex, non-linear feature spaces.

**Class (3) Optimization-Based Methods:** Optimization frameworks incorporate explicit spatial or operational constraints into the zoning process. Standard clustering may yield fragmented or irregular boundaries that are impractical for mechanized agricultural operations. To address this limitation, techniques such as integer linear programming (ILP), column generation, and bio-inspired Estimation of Distribution Algorithms (EDAs) have been applied to enforce geometric regularity and spatial contiguity constraints [136,137,143]. In this class, zoning is formulated as a constrained optimization problem balancing statistical homogeneity with operational constraints and feasibility.

**Class (4) Probabilistic/Evidential Methods:** Probabilistic and evidential approaches explicitly incorporate uncertainty into the zoning process. Rather than defining zones solely through geometric distance or variance minimization, these mechanisms model spatial units as realizations from underlying statistical distributions and assign probabilistic or belief-based measures of zone membership.

Gaussian Mixture Models (GMMs) exemplify this approach by representing field data as a mixture of latent distributions and estimating membership probabilities through maximum likelihood procedures [62]. Model selection criteria such as the Bayesian Information Criterion (BIC) are also applied to determine the optimal number of zones in a data-driven manner [18].

Evidential frameworks based on Dempster–Shafer theory, including the Transferable Belief Model, further extend this paradigm by enabling fusion of heterogeneous spatial datasets while explicitly representing belief uncertainty [83]. By producing probability distributions or quantified belief masses rather than strictly deterministic partitions, Class 4 methods provide an interpretable measure of confidence in zone assignments and facilitate integration of expert agronomic knowledge into the delineation process.

**Class (5) Rule-/Threshold-Based Methods:** Rule-based mechanisms delineate MZs by applying predefined statistical or agronomic thresholds to continuous spatial variables. Rather than discovering latent clusters, these approaches impose explicit boundaries based on quantiles, natural breaks, or expert-defined criteria. For instance, the Jenks Natural Breaks optimization is used to partition interpolated soil property maps into discrete MZs [144]. Thresholding strategies are also common in temporal stability mapping, where multi-year yield or VIs are categorized into stable–high, stable–low, and unstable classes [118,131]. Composite index frameworks similarly rely on fixed or semi-empirical cutoffs to translate continuous metrics into actionable MZs [145]. Class 5, therefore, derives zoning boundaries from externally defined decision rules rather than intrinsic similarity structure.

**Synthesis of Methodological Trends:** Across the reviewed literature, similarity-based clustering (Class 1) and rule-based thresholding (Class 5) remain the principal mechanisms for direct zone delineation. In contrast, machine learning, optimization-based, and probabilistic approaches (Classes 2–4) serve as specialized partitioning approaches to overcome the limitations of standard clustering. Specifically, they are utilized to map non-linear decision boundaries, enforce strict operational constraints, or mathematically incorporate expert knowledge and uncertainty directly into the zone delineation workflow. The methodological evolution of MZ delineation is therefore characterized less by replacement of traditional clustering than by its integration with advanced computational frameworks to improve spatial coherence, interpretability, and operational applicability. This pattern reflects a broader tendency in the field to retain simple and interpretable zoning mechanisms, while using advanced methods during data preparation such as feature construction, constraint formulation, and uncertainty modeling. The study-level coding used in this review (including workflow labels such as A1) is accessible through our interactive, web-based Review Database [49].

### 4.5. Context-Specific Delineation Strategies

Although many reviewed studies apply general-purpose zoning pipelines, MZ delineation is not a context-neutral task. The agronomic drivers of spatial variability differ across cropping systems, environmental conditions, and management objectives, and these differences influence which inputs, spatial units, and computational strategies are most appropriate.

Accordingly, a subset of studies use context-driven delineation strategies, in which feature selection, zoning logic, or spatial unit definition is explicitly adapted to crop physiology, dominant environmental limitations (e.g., water availability), or the intended agronomic intervention [2,55,139]. In some cases, algorithms are modified to account for machinery constraints [136,137]; in others, local farmer knowledge is directly integrated into the zoning process [83]. Taken together, these studies move beyond generic clustering paradigms and explicitly embed delineation design within defined agronomic and operational contexts.

This section synthesizes these context-specific adaptations. Although they represent a minority of the reviewed papers, they signal an emerging methodological shift in precision agriculture toward zoning frameworks embedded within defined agronomic contexts rather than treated as purely abstract spatial partitioning exercises.

#### 4.5.1. Crop-Specific Zoning Approaches

Delineation strategies vary significantly between arable crops and high-value perennial systems. For annual grain crops (e.g., maize, wheat, soybean), zoning typically relies on the integration of yield monitor data, topography, and soil sensors to capture yield stability over time [2,132]. In these systems, rotation effects, such as the soybean–corn sequence, introduce complexity, often requiring multi-year normalization or specific feature selection to account for different crop responses to the same soil properties [6,134]. Conversely, for high-value orchards [146] and vineyards, delineation shifts from continuous field mapping to object-based or individual-tree analysis. For instance, in almond and citrus orchards, LiDAR and UAV-based multispectral imagery are used to segment individual canopy volumes or leafiness indices to define zones for fertilization, treating the tree rather than the soil as the primary management unit [16,18]. Similarly, in viticulture, zoning often prioritizes vine vigor and water status stability to manage fruit quality and maturation uniformity [55,77]. These examples show that the effective decision unit of MZ delineation shifts with crop architecture and management scale: annual systems are typically zoned as continuous field surfaces, whereas perennial systems more often require plant- or row-level structural characterization.

#### 4.5.2. Environmental and Regional Adaptations

The MZ delineation workflow is strongly dictated by the region’s primary limiting factor, particularly water availability. In rainfed systems, where water redistribution drives yield, topography and soil depth are often the dominant weighting factors for zoning algorithms [2,99]. In these environments, hydrologic classification frameworks (e.g., Budyko) have been adapted to identify sub-field zones prone to runoff or leaching [99]. In contrast, for irrigated arid regions, zoning strategies focus heavily on soil salinity and hydraulic properties (e.g., water-holding capacity) to optimize variable rate irrigation [50,69,147]. Papers in these contexts often prioritize $EC_{a}$ sensing and thermal imagery (e.g., CWSI) to delineate MZs that specifically reflect crop water stress and salinity tolerance rather than general fertility [50,69,93]. Furthermore, regional economic constraints influence methodology; in smallholder farming systems where machinery data is scarce, delineation relies heavily on accessible remote sensing platforms (such as satellite imagery or low-cost UAVs) and sparse sampling rather than sensor-fusion [102,126]. Overall, these studies show that regional constraints do not simply affect the availability of data; they also reshape the logic of delineation itself by changing which stressors must be represented and which sensing strategies remain operationally feasible.

#### 4.5.3. Objective-Driven Workflows

The practical implementation of MZ delineation is inherently tied to a specific agronomic objective. To systematically examine the relationship between delineation strategies and management goals, we identified studies that explicitly defined a management objective such as fertilization, irrigation, seeding, pest control, or soil remediation. Papers that treated MZ delineation solely as a general site-specific management framework, without linking zones to a specific intervention, were excluded from this objective-specific classification.

Table 2 catalogs the subset of papers in which the intended agronomic intervention was clearly defined. Analysis of these objective-driven workflows reveals that the selection of input variables, spatial constraints, and computational logic varies systematically with management purpose.

For nutrient management, particularly variable-rate fertilization, zoning frameworks typically emphasize relatively stable soil chemical and physical properties, including nutrient distributions, SOM, and long-term yield potential [148]. These workflows frequently employ fuzzy clustering (e.g., Fuzzy c-Means) to partition fields into discrete fertility zones that align nutrient inputs with localized soil variability [64,135,141,149].

In contrast, zoning for variable-rate seeding centers on identifying spatially distinct regions with different yield potential. High-productivity zones receive higher planting densities, whereas resource-constrained areas receive lower planting densities to mitigate overcrowding. These delineation strategies rely heavily on multi-year yield stability analysis, topographic indices, and population optimization models that estimate localized carrying capacity [17,22,67,96].

The need for responsive in-season management has introduced a shift toward dynamic delineation, particularly for precision irrigation. Because static soil hydraulic zones may fail to capture intra-season variability in crop water demand, irrigation-oriented workflows increasingly integrate temporal remote sensing indicators, such as ET, crop water stress index, and canopy temperature, alongside real-time soil moisture sensing. This enables adaptive, time-specific adjustment of MZs boundaries throughout the growing season [89,93,94,103,112].

Soil remediation and hydrologic management objectives aim to mitigate severe physical or chemical constraints that limit crop performance rather than maximizing productivity or input efficiency. In salt-affected regions, delineation frameworks rely heavily on apparent electrical conductivity ($EC_{a}$) sensing and remote sensing indicators to map root-zone salinity, enabling targeted salt leaching and variable-rate application of soil amendments such as gypsum [69,140,150]. Conversely, drainage- and runoff-oriented workflows integrate terrain derivatives (e.g., topographic wetness index) and spatially explicit hydrologic models, to identify sub-field areas prone to saturation, runoff accumulation, or excessive leaching [99]. In these contexts, zoning serves environmental conservation and risk mitigation purposes rather than direct yield enhancement.

Finally, pest and weed management workflows differ fundamentally from productivity-oriented zoning. Rather than optimizing yield inputs, these approaches incorporate biotic stress indicators, species distribution modeling, and object-based image classification to identify localized infestation patches. In this context, zones are delineated explicitly for targeted agrochemical intervention and ecological containment [98,139].

Collectively, these results show that once MZ delineation is anchored to a specific agronomic objective, feature selection, spatial constraints, and workflow design become more targeted, moving zoning from generic spatial partitioning toward intervention-specific decision support.

**Table 2:** Classification of reviewed studies based on explicitly disclosed agronomic management objectives. Studies treating MZ delineation as a generic spatial problem without a specific input application were excluded from this classification.

Explicit Management Focus	Included Studies
Nutrient Management * (Variable Rate Fertilization, Nitrogen/Phosphorus Management)	[16,17,20,64,65,79,100,111,124,135,141,142,149,151,152,153,154]
**Variable Rate Seeding** (Planting Density Optimization)	[17,22,67,96]
**Precision Irrigation** (Variable Rate Irrigation, Water Stress Management)	[2,19,50,87,93,94,103,106,112,144,155,156,157]
**Soil Remediation & Hydrology** (Salinity Mitigation, Gypsum Application, Drainage & Runoff Control)	[69,99,140,150]
**Pest & Weed Management**	[98,139]

* Bold text indicates the agronomic management categories, while the text in parentheses provides application examples.

### 4.6. Validation and Evaluation Approaches

**Figure 8:**
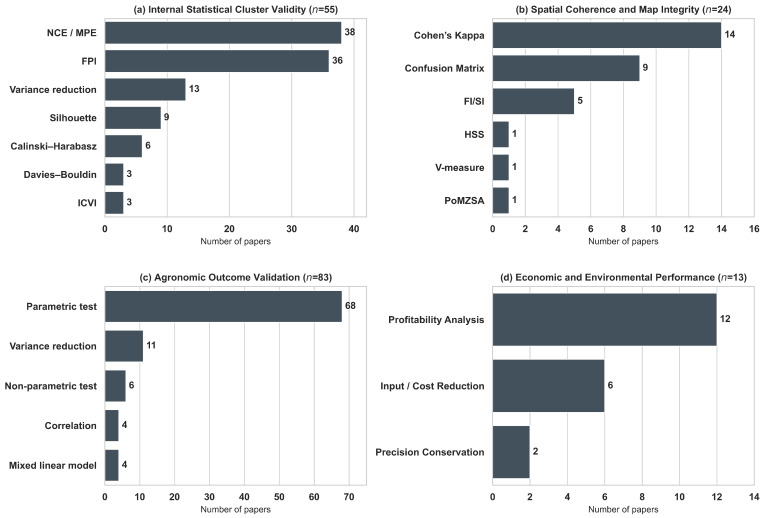
Validation and evaluation approaches used to assess delineated MZs across the reviewed papers. Each panel summarizes one validation tier: (**a**) internal statistical validation, (**b**) spatial coherence and agreement, (**c**) agronomic outcome validation, and (**d**) economic and environmental performance assessment. Bars indicate the number of papers reporting each metric or evaluation approach. A single study may report multiple validation methods within or across tiers; therefore, bar totals may exceed the panel-specific sample size. The value *n* in each panel denotes the number of papers employing at least one method within that validation tier, based on the final extracted dataset.

The reviewed literature reveals a layered but uneven validation structure for MZ delineation (Figure 8). Most papers assess zoning quality using internal statistical criteria during or immediately after clustering, whereas fewer extend evaluation to spatial coherence, agronomic response, or economic and environmental performance. This pattern indicates that validation in MZ research remains focused on statistical optimality rather than practical effectiveness. To clarify how validation is currently conducted, the reported approaches are organized here into four tiers: (i) internal statistical cluster validity, (ii) spatial coherence and map integrity, (iii) agronomic outcome validation, and (iv) economic and environmental performance. Because multiple validation approaches can be applied within a single study, individual papers may contribute to more than one tier or metric count. While this framework captures the predominant validation strategies reported in the reviewed papers, alternative evaluation paradigms that do not fit cleanly within these tiers are discussed separately at the end of this section.

#### 4.6.1. Internal Statistical Cluster Validity

The first type of evaluation occurs during the clustering process to determine the optimal number of clusters. For fuzzy clustering methods (e.g., FCM), the most widely used metrics are the Fuzziness Performance Index (FPI) and Normalized Classification Entropy (NCE) or Modified Partition Entropy (MPE) [6,21,140]. The FPI estimates the degree of membership sharing among clusters, whereas NCE and MPE measure the degree of disorganization. Minimizing these indices is the standard protocol for identifying the optimal number of clusters [5,140]. For hard clustering approaches (e.g., K-means, Hierarchical), the Silhouette Index, the Davies–Bouldin Index, and the Calinski–Harabasz (CHI) Index are widely used to maximize intra-cluster compactness and inter-cluster separation [62,74]. More recently, composite indices such as the Improved Cluster Validation Index (ICVI) have been proposed to integrate multiple internal metrics into unified decision frameworks, thereby reducing reliance on a single statistical criterion [109].

While these internal measures are useful for identifying statistically compact and well-separated clusters, they do not establish whether the resulting zones are spatially practical or agronomically meaningful, necessitating additional validation layers.

#### 4.6.2. Spatial Coherence and Map Integrity

Following statistical partitioning, the spatial structure of delineated zones must be evaluated to ensure positional consistency and operational feasibility. A primary concern is excessive fragmentation, commonly referred to as the salt-and-pepper effect, which makes the zones harder to use with agricultural machinery and variable-rate application systems. To improve spatial contiguity, post-processing techniques such as median filtering, morphological opening and closing, and boundary smoothing are applicable [108]. The effectiveness of these procedures is assessed using structural metrics such as the Smoothness Index (SI) and Fragmentation Index (FI), which quantify the spatial frequency of class transitions and ensure compliance with minimum area or shape constraints [68].

Beyond geometric coherence, map integrity is evaluated through categorical agreement analysis between delineated MZs and reference classifications (e.g., yield-based zones or expert-defined units). Cohen’s Kappa coefficient is widely used to measure agreement beyond random chance, while confusion matrices provide overall, user’s, and producer’s accuracy metrics [71,88]. However, Kappa assumes independence among spatial units and may be sensitive to class imbalance in heterogeneous fields. To address these limitations, advanced spatial concurrence metrics have been adopted, including the Heidke Skill Score (HSS) to strictly control for random spatial chance [3], the V-measure to evaluate spatial pattern similarities between alternative cluster maps [16], and the Percentage of Management Zones Spatial Agreement (PoMZSA) for quantifying exact spatial overlap [158].

Together, these metrics assess whether statistically optimal clusters result in spatially coherent and positionally credible maps. This tier, therefore, acts as the bridge between statistically derived clusters and operationally usable zone maps.

#### 4.6.3. Agronomic Outcome Validation

Because one of the main purposes of MZ delineation is to differentiate areas with distinct productivity or response potential, external agronomic validation constitutes a critical third tier. Historical yield maps frequently serve as the reference benchmark. The most common metric is Variance Reduction (VR), which calculates the percentage of total field yield variability explained by the delineated zones [108]. To statistically verify that zones represent distinct yield potentials, Analysis of Variance (ANOVA) is frequently used [23,158]. However, due to the spatial autocorrelation inherent in field data, standard ANOVA violates the assumption of independent observations. Consequently, spatial Mixed Linear Models (MLM) or non-parametric tests (e.g., Kruskal–Wallis) are increasingly recommended to compare zonal means while accounting for spatial structure [58,69]. Beyond yield, zones are validated against soil nutrient samples to ensure that the delineation captures underlying fertility gradients [66].

Furthermore, correlation analyses (e.g., Pearson’s *r*) are employed to quantify continuous relationships between delineated zones and agronomic targets such as yield gradients or soil nutrient concentrations [92,121].

Whereas spatial agreement metrics evaluate positional consistency between categorical maps, agronomic validation methods assess whether delineated zones correspond to statistically distinct or continuously varying field responses. This tier, therefore, determines whether zoning boundaries reflect meaningful biological and production differences rather than merely statistical segmentation.

#### 4.6.4. Economic and Environmental Performance

The final tier evaluates whether delineated zones translate into tangible managerial value. However, only a limited subset of the reviewed literature (13 papers) explicitly extends validation to economic or environmental performance. Gross margin and net return analyses are utilized to determine whether site-specific management increases farm profitability compared to uniform management [82,131,154]. Several studies report that input optimization based on MZs, such as reducing nitrogen in unstable or low-yield zones or increasing seeding rates in high-potential zones, can improve partial economic returns [96,155]. From a sustainability perspective, precision conservation approaches validate zones by identifying low-profit or environmentally sensitive areas (e.g., eroded slopes or saline spots) that may be better managed for biodiversity or conservation rather than yield maximization [3,131].

Taken together, this tier assesses whether statistically sound and agronomically differentiated zones translate into financial or environmental benefits. At the same time, its limited representation in the literature (Figure 8d) suggests that MZ studies still prioritize statistical and agronomic differentiation more often than fully validated management outcomes. This gap likely occurs because proving real management value requires extra time, economic data, and multi-season field evidence.

#### 4.6.5. Alternative Evaluation Paradigms

Although most reviewed studies can be interpreted within the four-tier framework above, a smaller subset adopts evaluation paradigms that do not fit cleanly within these categories.

Research focused on continuous spatial regression or process-based crop modeling often bypasses discrete boundary validation in favor of continuous predictive error metrics, such as Root Mean Square Error (RMSE), Mean Absolute Error (MAE), and the coefficient of determination ($R^{2}$), to evaluate how accurately the models predict yield or soil parameters at the pixel level [53,75,113,157]. Conversely, studies introducing novel computational methods for zone delineation, such as bio-inspired optimization or integer linear programming, frequently define success based on algorithmic efficiency, computational execution time, and mathematical variance rather than in-field agronomic reality [136,137,143]. Finally, a few studies that present new spatial decision support systems (SDSS) or open-source framework architectures rely on qualitative system descriptions rather than on formal statistical zone validation [4,114,123].

These alternative paradigms show that MZ delineation is not always evaluated as a field decision-support problem; in some studies, it is assessed primarily as a predictive, algorithmic, or software-development task.

## 5. Discussion

The synthesized literature demonstrates a clear methodological transition in MZ delineation: early research emphasized static soil- or yield-based partitioning, whereas contemporary research reflects increasing methodological sophistication, expanding data integration, and growing sensitivity to field-scale heterogeneity and agronomic objectives. At the same time, the review reveals that validation practices—particularly those linking delineation outcomes to agronomic and economic performance—remain unevenly developed across the literature.

This section synthesizes the findings across four interrelated dimensions. First, we examine the geographic distribution of studies and the implications of field-scale heterogeneity across diverse agro-climatic regions (Section 5.1). Second, we analyze how cropping systems and management objectives influence delineation logic and decision criteria (Section 5.2). Third, we evaluate prevailing data–method combinations and emerging computational trends (Section 5.3). Finally, we identify remaining challenges, research gaps, and future opportunities that shape the trajectory of MZ delineation research (Section 5.4).

### 5.1. Geographic Distribution and Field Scale Heterogeneity

A clear contrast in field size emerges between the Americas and Eurasia. Papers originating from Brazil and the United States frequently address large-scale, mechanized commercial operations. For instance, MZs are delineated in soybean and cotton production systems in Brazil, ranging from 150 to over 260 ha [102,134]. In these contexts, the primary challenge is managing extensive spatial variability where high-density data is abundant but computational efficiency is critical [74]. Conversely, research from Asia and parts of Europe often focuses on smallholder or fragmented landscapes. In Pakistan, precision zoning is applied to wheat fields as small as 0.4 ha [153], while in China, researchers emphasized the optimization of zoning scales to accommodate the transition from household plots to cooperative management [81]. This difference requires different algorithmic approaches; while large-scale studies prioritize filtering and dimensionality reduction of massive datasets [74,120], small-scale studies often rely on intensive sampling or high-resolution proximal sensing to capture micro-variability [52,159]. This scale-dependent contrast highlights a fundamental methodological challenge: the lack of universal, multi-scale frameworks capable of balancing the computational efficiency needed for large-scale operations with the high-resolution sensitivity required for fragmented smallholder landscapes. Advancing MZ delineation, therefore, requires scalable, multi-source workflows that can adapt to heterogeneous field sizes without sacrificing statistical robustness or operational feasibility. Addressing this imbalance is critical for reducing the structural disparities in technology adoption observed across regions and for improving the transferability of delineation strategies across global production systems.

### 5.2. Crop-Specific and Objective-Driven MZ Delineation

The specific management objectives driving MZ delineation vary significantly across agro-ecological contexts, reflecting differences in climatic constraints, crop physiology, and production intensity.

In temperate and subtropical rainfed grain systems, such as the U.S. Midwest, Southern Brazil, and Northern Europe, the dominant objective remains nutrient optimization. Here, delineation strategies primarily target nitrogen, phosphorus, and potassium management to enhance yield stability within corn, wheat, and soybean rotations [23,119]. Increasingly, nutrient MZs are evaluated not solely by yield response but by economic indicators such as gross margin stability, reflecting a shift toward profitability-based delineation criteria [131].

In contrast, arid and semi-arid regions, including California, Israel, and Xinjiang (China), prioritize water-use efficiency and salinity mitigation. In these environments, MZ delineation increasingly adopts dynamic frameworks in which zones are updated intra-seasonally using thermal and spectral indicators (e.g., CWSI, ET) to capture transient water stress conditions [93,94]. Time-series remote sensing has also been integrated to manage spatial salinity gradients in crops such as cotton [81], illustrating a transition from static soil-based zoning toward temporally responsive water and salinity management.

High-value perennial systems, including orchards (olive, almond, citrus) and vineyards, particularly in Mediterranean climates and California, introduce a distinct paradigm in which canopy geometry becomes a primary determinant of spatial variability [16,18]. Rather than centring delineation on soil-property gradients, these workflows incorporate explicit geometric descriptors of plant architecture. Structural traits such as individual tree height, canopy volume, and canopy height profiles, derived from UAV-based surface models or LiDAR reconstructions, are used as geometry-informed inputs to clustering algorithms [16]. In super-intensive almond systems, geometric canopy descriptors, including height models and cross-sectional measures of leafiness and porosity, define vigor classes aligned with canopy functioning and light-interception capacity [18,125]. In this objective setting, MZs are therefore anchored to structural canopy heterogeneity, enabling interventions such as pruning, canopy regulation, and fertigation to be aligned with spatial variation in physical canopy capacity and light interception, rather than being inferred indirectly from soil maps alone [18,125].

Beyond input optimization, emerging research expands MZ applications toward long-term sustainability objectives. Integrated Crop–Livestock Systems (ICLS), for example, require crop-specific delineation to accommodate differing pasture and cash-crop requirements [6]. Similarly, recent studies have delineated zones based on soil carbon stock variability, reflecting growing interest in ecosystem service valuation and soil health monitoring within precision agriculture frameworks [151].

Across these systems, a broader methodological transition is evident: the movement from static to dynamic zoning paradigms. Traditional approaches assume relatively stable yield-limiting factors over time [63,100]. However, recent findings suggest that static zones often fail to capture intra-seasonal variability driven by weather and short-term nutrient availability. Time-specific MZ delineation frameworks integrating plant and soil sensing have therefore been proposed to address the spatiotemporal dynamics of crop–environment interactions [89]. The increasing availability of high-frequency satellite imagery (e.g., Sentinel-2) further enables recalculation of MZ boundaries at critical phenological stages, such as peak biomass during variable-rate nitrogen application [3,134].

Despite these advancements, several critical gaps remain. First, rotation complexity remains insufficiently addressed. Most MZ frameworks are optimized for single crops, yet rotational systems (e.g., soybean–corn or crop–pasture sequences) exhibit crop-specific responses to identical soil constraints, requiring multi-crop feature selection strategies to avoid conflicting management boundaries [6,156]. Second, the integration of static and dynamic zoning paradigms remains unresolved, while intra-season MZs capture temporary weather and water stress, high-frequency data acquisition can reduce net profitability [94]. Future research should prioritize hybrid frameworks in which stable, soil-derived baseline zones are selectively adjusted using temporal remote sensing at critical phenological stages [89,103]. Third, there is still a gap between what works best statistically and what is practical in the field. Conventional clustering often produces fragmented boundaries that are incompatible with standard variable-rate machinery. Incorporating geometric and contiguity constraints within optimization-based zoning frameworks is therefore essential to ensure field-level practical feasibility [136,137]. Fourth, validation protocols lack standardization, as there are currently no universally accepted guidelines or analytical protocols for establishing and evaluating potential MZs [56]. Many studies rely primarily on internal clustering indices, whereas rigorous evaluation requires independent agronomic and economic assessment, including multi-year yield stability and gross margin analysis [82,131]. Finally, future MZ research should extend beyond short-term yield maximization toward sustainability-oriented objectives, including soil carbon management and ecosystem service optimization [131,151].

### 5.3. Data and Workflows, Combinations and Trends

The review period (2000–2025) captures a pronounced technological transition in MZ delineation. Early studies predominantly relied on yield maps and sparse soil sampling processed through standard clustering techniques (e.g., k-means) [71,84,140]. These workflows largely assumed temporal stability and emphasized static, soil-driven partitioning. In contrast, literature from the last five years (2020–2025) reflects increasing methodological complexity, driven by data fusion and advanced learning frameworks. Recent studies integrate satellite time-series, proximal sensing, terrain derivatives, and ensemble learning methods (e.g., Random Forest, stacking) to construct higher-dimensional representations prior to zoning [23,81,104]. This shift signals a movement from single-layer clustering toward multi-source, feature-engineered delineation pipelines [60,74].

Parallel to this data integration trend, the long-standing assumption of temporal stability has been progressively reconsidered. Rather than treating MZs as fixed soil-based entities, recent studies propose dynamic zoning frameworks that adapt to seasonal climate variability and crop phenological stages [94,112]. Consequently, delineation has evolved from permanent spatial partitions toward functional, time- and decision-specific MZs [89,103].

**Figure 9:**
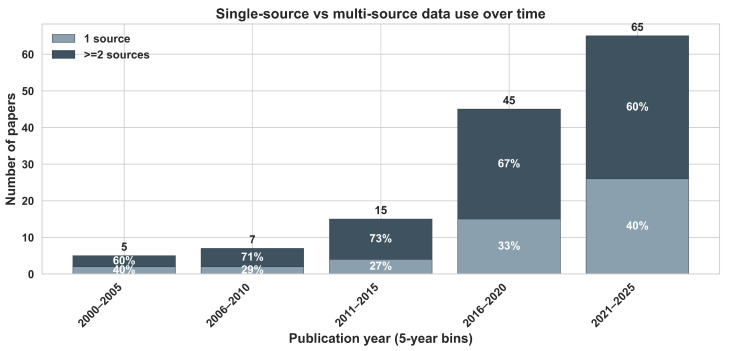
Temporal trends in single-source vs. multi-source data use for MZ delineation (2000–2025). Bars represent total publications within five-year intervals, partitioned by data-source category.

Figure 9 quantifies this transition by illustrating the distribution of single-source and multi-source workflows across five-year intervals. Multi-source approaches consistently account for the majority of published studies. Although their proportional dominance remains relatively stable, the absolute number of multi-source studies increases markedly after 2015, coinciding with expanded access to satellite time-series products, proximal sensing technologies, and high-resolution terrain datasets [73].

Importantly, single-source studies have not disappeared but remain alongside data-fusion workflows. This coexistence indicates that increasing data integration has expanded the methodological landscape without eliminating lower-complexity zoning frameworks, which remain operationally relevant due to data availability, cost, or computational resources [63,134].

An additional pattern emerging from this transition concerns validation practices, while methodological complexity and data integration have increased substantially, evaluation strategies have not evolved at the same pace. Most workflows continue to rely on statistical or spatial agreement metrics, with comparatively fewer studies extending validation toward agronomic outcomes or economic performance. This imbalance suggests that many MZ delineation pipelines focus on statistical consistency rather than proving their practical value in the field.

**Figure 10:**
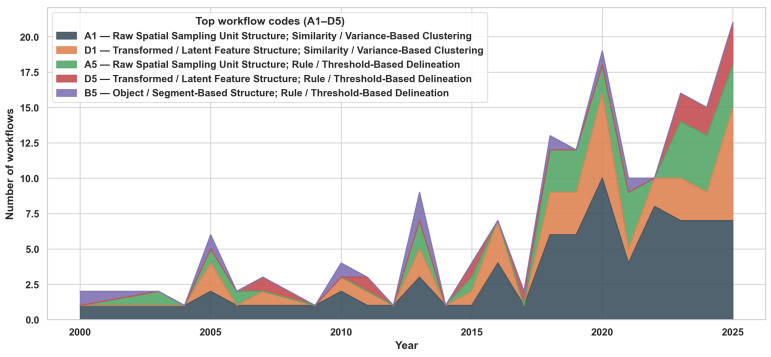
Temporal evolution of the top five MZ delineation workflow codes (A1, D1, A5, D5, B5) from 2000 to 2025. The stacked areas represent the annual number of published studies implementing each workflow, illustrating both the overall increase in research activity and the diversification of delineation strategies over time.

**Figure 11:**
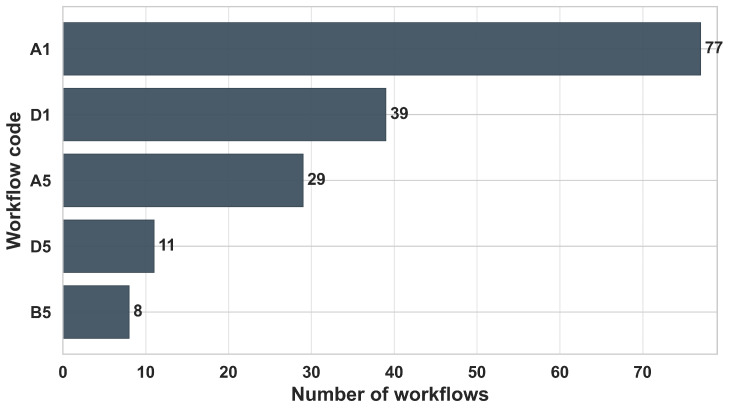
Top five most frequently reported MZ delineation workflows, as defined in Section 4.4. Bars indicate the number of papers implementing each specific workflow. The workflow code key is provided within the figure. Similarity-/variance-based clustering emerges as the dominant zoning mechanism, most frequently applied to raw spatial sampling units (A1) and, secondarily, to transformed/latent feature structures (D1).

Beyond the temporal growth documented in Figure 10, the structural combinations illustrated in Figure 11 reveal important patterns in how data representation (Dimension 1) and zoning mechanisms (Dimension 2) interact. Rather than operating as isolated algorithmic choices, contemporary MZ workflows are increasingly structured as multi-stage analytical pipelines.

Figure 11 presents the five most frequent workflows identified in the reviewed literature: A1, D1, A5, D5, and B5. The dominance of A1 and D1 configurations indicates that similarity/variance-based clustering (class 1) remains the core partitioning engine. However, the associated feature representation has progressively evolved—from raw spatial sampling units (A) toward transformed, reduced, or latent feature spaces (D). This pattern suggests that recent methodological innovation has focused more on enhancing data representation and feature engineering than on replacing the underlying clustering paradigm itself. Meanwhile, the frequent emergence of A5, D5, and B5 workflows illustrates a parallel diversification, with rule- or threshold-based mechanisms (Class 5) being applied across raw (A), object-based (B), and transformed (D) data structures.

Consequently, recent studies frequently integrate classical clustering with spatial transformations and dimensionality reduction models [23,58,62,74,87]. Such hybridization of methods reflects a clear trend toward modular, compositional workflows in which multiple analytical operators are combined to balance accuracy, spatial coherence, and operational feasibility.

**Figure 12:**
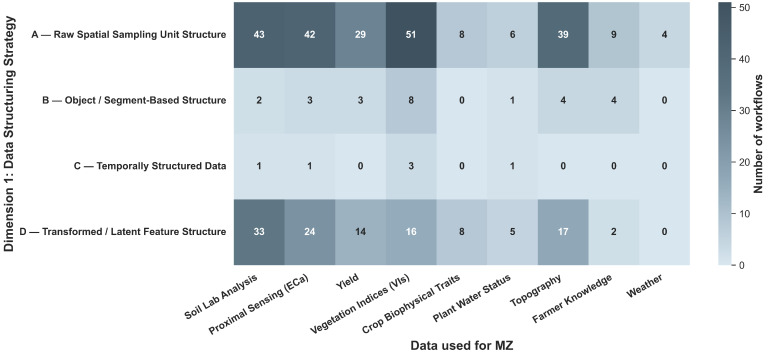
Co-occurrence frequency between data types and Dimension 1 structuring strategies (Raw Spatial Sampling Unit, Object/Segment-Based, Temporally Structured, and Transformed/Latent Feature). Cell intensity reflects the number of papers reporting each data–representation pairing.

The heatmap in Figure 12 highlights clear representation patterns across data types. Raw spatial sampling unit structuring overwhelmingly dominates soil laboratory analysis, proximal sensing ($EC_{a}$), VIs, yield maps, and topographic derivatives, confirming that grid-based representations remain the foundational architecture of MZ delineation. Transformed or latent feature structures constitute the second most frequent strategy, particularly for soil and proximal sensing inputs, indicating that recent methodological development has prioritized feature engineering and dimensionality reduction rather than abandoning raw spatial units. In contrast, object-/segment-based and explicitly temporally structured representations remain comparatively limited, with object-based approaches largely confined to vegetation imagery and temporally structured workflows still sparsely adopted despite increasing interest in dynamic zoning.

**Figure 13:**
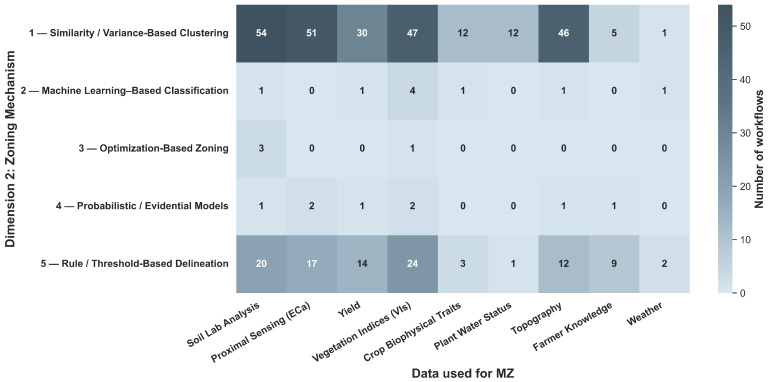
Co-occurrence frequency between computational method classes and data types across the reviewed literature. Cell intensity reflects the number of studies reporting each specific method–data pairing.

The heatmap in Figure 13 reveals a similarly structured pattern at the mechanism level. Similarity-/variance-based clustering shows consistently high co-occurrence across nearly all major agronomic descriptors, reinforcing its role as the dominant partitioning engine of the field more likely due to its simplicity. Rule-/threshold-based delineation appears as a broadly distributed secondary mechanism, particularly for VI analyses where predefined agronomic thresholds enable straightforward interpretation. In contrast, machine learning-based classification, optimization-based zoning, and probabilistic/evidential approaches remain comparatively concentrated and context-specific, suggesting that while methodological diversification has expanded, the core delineation paradigm continues to rely primarily on clustering-based partitioning applied to foundational soil–crop–terrain variables.

Despite the increasing sophistication of MZ delineation workflows, several structural and methodological limitations remain evident. First, as multi-source and multi-year data fusion becomes standard practice, robust temporal validation and anomaly management remain underdeveloped. The widespread aggregation of historical yield maps and satellite time-series implicitly assumes that multi-year averaging captures intrinsic field potential. However, extreme climatic events, localized pest outbreaks, and sensor artifacts can introduce historical anomalies that distort zone boundaries. Future research will require the integration of systematic outlier detection and data-selection protocols within zoning pipelines to ensure that delineations reflect stable agronomic patterns rather than short-term environmental noise [120]. Second, although clustering remains the dominant partitioning paradigm, increasing reliance on transformed and high-dimensional feature spaces introduces concerns about interpretability and agronomic transparency. When zoning is performed on latent or composite features, the physical meaning of zone boundaries may become obscured. Developing explainable frameworks, whether through feature attribution methods, stability analysis, or agronomically constrained modeling, will be critical to ensure that highly accurate models are actually trusted and used by farmers [23]. Third, the consolidation toward hybrid, multi-stage analytical pipelines raises issues of scalability and accessibility. Contemporary workflows often require advanced geospatial preprocessing, feature engineering, and computational resources that exceed the technical capacity of many agricultural stakeholders. To address the computational barriers associated with hybrid, multi-stage analytical pipelines, the recent literature highlights the need to integrate automated data harmonization and zoning protocols into open-source, cloud-based Spatial Decision Support Systems (SDSSs) that do not require local high-performance computing [116,123]. These limitations motivate the broader set of challenges and research opportunities discussed in the following section.

### 5.4. Challenges, Research Gaps and Opportunities in MZ Delineation

Our analysis of geographic patterns, data structuring strategies, and workflows in the reviewed papers reveals several cross-cutting challenges that shape the future trajectory of MZ delineation research.

#### 5.4.1. Data Robustness and Multi-Source Integration

There is a constant challenge to balance having more data with making sure that data is reliable, while multi-source and multi-year data fusion has become standard practice, sensitivity to sensor choice, cloud contamination [160], temporal reconstruction, and historical anomalies [120] remains insufficiently addressed. Substituting satellite imagery with UAV data, for example, can substantially alter zone boundaries, underscoring the need for standardized preprocessing, cloud-detection, and spatiotemporal reconstruction protocols to ensure reproducibility and robustness across sensing platforms [127]. Future frameworks should incorporate systematic anomaly filtering and uncertainty quantification to prevent temporary environmental noise and abnormal events from propagating into long-term zoning decisions, thereby mitigating the risk of misleading zone boundaries [120].

#### 5.4.2. Methodological Sophistication and Accessibility

The field struggles to balance complex methods with practical, easy-to-use tools. As workflows evolve toward hybrid, multi-stage pipelines involving dimensionality reduction, ensemble learning, and spatial optimization, the technical and computational requirements increasingly exceed farm-level capacity. Without scalable, cloud-enabled Spatial Decision Support Systems (SDSSs) and modular open-source implementations, advanced delineation strategies risk widening the digital divide between technologically equipped large-scale operations and resource-constrained producers [123].

#### 5.4.3. Static vs. Dynamic Zoning Paradigms

The coexistence of static and dynamic paradigms reflects an unresolved conceptual gap, while dynamic zoning captures intra-seasonal variability driven by climate and phenology, static soil-based zones remain operationally simpler and economically stable [94]. Developing hybrid frameworks that anchor delineation in stable structural soil features while adaptively modulating prescriptions at critical phenological thresholds represents a promising research direction capable of reconciling robustness with responsiveness [93].

#### 5.4.4. Statistical Optimality vs. Operational Feasibility

A gap remains between what is statistically optimal and what is practical in the field. Fragmented or highly irregular cluster outputs often conflict with machinery constraints and real-world application logistics. Integrating geometric constraints, minimum area thresholds, and contiguity rules directly into zoning algorithms, rather than as post hoc smoothing operations, remains essential for translating computational delineations into actionable field prescriptions [137].

#### 5.4.5. Inconsistent Validation Practices

Validation practices remain inconsistent and are frequently limited to internal clustering indices or spatial agreement measures. While these metrics assess statistical coherence, they do not necessarily demonstrate whether delineated zones translate into meaningful agronomic, economic, or environmental outcomes. Only a limited subset of studies extends validation to field-based performance indicators such as input-use efficiency or gross margin analysis. The limited extent of economic validation remains a key challenge. Uncertain financial returns and the risk of economic losses from poorly designed MZs continue to constrain the wider adoption of variable-rate technologies [22,82]. This imbalance reflects the practical challenges of collecting multi-season agronomic data and detailed economic records required for a comprehensive evaluation. Furthermore, comparing profitability across studies is difficult due to varied experimental setups, fluctuating chemical prices, and differing assumptions about which operational costs, such as spatial data acquisition or new machinery, are included in the analysis [154]. Advancing MZ delineation toward operational decision-support systems will therefore require standardized evaluation protocols that combine statistical metrics with agronomic field trials and economic performance assessment [22]. Strengthening this link between computational outputs and real-world outcomes is essential to demonstrate not only spatial coherence but also tangible profitability and sustainability benefits [82].

#### 5.4.6. Emerging Directions Beyond the Review Scope

Despite the comprehensive scope of this paper, MZ delineation remains a rapidly evolving domain in which new conceptual directions continue to emerge. Recent papers outside the reviewed papers further underscore emerging directions. For example, multi-year, multi-sensor VIs composites to capture long-term spatial stability enable delineation based on persistent agricultural potential [161]. Human-in-the-loop visual analytics approaches emphasize terrain-aware refinement by explicitly integrating landform classifications derived from DEMs into delineation pipelines [11]. In parallel, the deployment of agentic AI frameworks promises to enhance autonomous, context-aware decision-making and resource optimization, but it also introduces practical challenges, including high infrastructure costs and data privacy concerns [162]. Moreover, emerging phytosanitary applications extend MZ frameworks beyond fertility optimization toward disease-risk mapping using multi-temporal soil and VIs [163]. Additionally, agricultural digital twin frameworks are being explored to optimize sensor deployment and sampling design by fusing remotely sensed data for irrigation MZ delineation [164]. These developments suggest that MZ delineation is progressively expanding toward long-term temporal integration, terrain-contextual modeling, crop-health-oriented decision support, and predictive digital-twin-assisted sensing strategies. Accordingly, while the taxonomy proposed herein captures dominant methodological patterns, it should be viewed as an evolving framework adaptable to future innovations.

Taken together, these challenges indicate that the next generation of MZ research should shift its emphasis from continued algorithmic development toward improved robustness of multi-source and multi-temporal data, standardized feature extraction, model interpretability, workflow scalability, and the systematic integration of validation across statistical, agronomic, and economic levels. To turn MZ delineation from a research exercise into a widely deployable tool aligning technical innovation with agronomic applicability and practical implementation constraints is essential.

## 6. Conclusions

This systematic review of 137 papers demonstrates that most operational workflows have moved away from single-variable clustering; instead, they increasingly fuse static soil indicators (e.g., $EC_{a}$ or topography) with dynamic crop phenology data (e.g., time-series NDVI) to address localized yield-limiting factors such as water stress or nutrient deficiency [58,91]. At the same time, the review shows that validation practices have not kept pace, with most studies still relying on internal statistical metrics rather than comprehensive agronomic or economic assessments. Methodologically, while Fuzzy c-means remains one of the most widely used methods due to its ability to handle gradual soil transitions, there is a clear trend toward spatially constrained clustering and bio-inspired optimization algorithms that prioritize operational feasibility over purely statistical homogeneity [5,137].

Despite methodological advancements, the “digital divide” remains a significant implementation barrier; complex deep learning models and high-dimensional datasets are often out of reach for smallholder farmers due to constraints in computational infrastructure and technical expertise. To bridge this gap, future research should prioritize the development of open-source, cloud-based decision support systems, such as those leveraging Google Earth Engine or web-based architectures, that democratize access to advanced zoning algorithms by eliminating the need for specialized local hardware. Ultimately, the widespread adoption of MZ technology relies on transitioning the research focus from algorithmic novelty to rigorous agronomic and economic validation, demonstrating that site-specific management delivers tangible, sustainable profits and resource conservation across diverse cropping systems.

Although this systematic review synthesizes 137 peer-reviewed papers, several limitations should be acknowledged. First, the analysis is restricted to peer-reviewed journal articles indexed in major scientific databases and further constrained to higher-impact (Q1-ranked) venues. While this restriction enhances methodological consistency and quality control, it inevitably introduces a selection bias. Specifically, it may underrepresent non-Q1-published papers, conference proceedings, technical reports, extension publications, and innovative practices published in lower-tier or non-indexed outlets. As a result, the synthesized trends primarily reflect the methodologically mature segment of the field and may not fully capture emerging practices, region-specific adaptations, or implementations in resource-constrained agricultural systems. In addition, the focus on peer-reviewed literature may bias the sample toward studies reporting statistically significant or technologically advanced outcomes. Furthermore, the threshold-based screening and ranking procedure introduces a potential risk of omissions (false negatives), particularly near selection boundaries. Nevertheless, given the rapid growth of the literature, exhaustive manual screening of all retrieved records is increasingly impractical. In this context, technology-assisted prioritization provides a pragmatic balance between feasibility and coverage, as discussed by Saeidmehr et al. [37], enabling efficient reduction of screening workload while retaining the most relevant studies.

Second, although paper screening and classification were assisted by LLMs to enhance consistency and scalability, all categorizations were manually reviewed. Nonetheless, classifying data and methods into specific categories inevitably involves some interpretive subjectivity. Third, substantial heterogeneity exists across studies in terms of field size, crop type, sensor resolution, and validation metrics, limiting direct quantitative comparability. Finally, given the rapid pace of methodological development—particularly in machine learning and cloud-based platforms—recent advances published after October 2025 may not be captured within the present reviewed literature.

Despite these constraints, this paper provides a structured synthesis of the methodological development of MZ delineation research from 2000 to 2025 and establishes a transparent framework for future comparative analysis.

Building on the identified limitations of this systematic review, future research should expand the evidence base beyond Q1-ranked journals to include conference proceedings, technical reports, and practice-oriented publications, thereby capturing a broader spectrum of real-world implementations. Furthermore, to reduce human interpretive subjectivity and improve consistency when categorizing complex or hybrid methodologies, future systematic reviews could leverage advanced methods, such as transformer-based document embeddings and density-based clustering techniques [165].

Ultimately, several avenues for future work stem directly from the research gaps identified in this article, specifically, addressing the substantial heterogeneity in field conditions, crop types, and validation metrics requires developing standardized reporting and evaluation protocols to enable more consistent, comparable assessments across studies and to ensure that delineated zones translate into measurable profitability and resource efficiency. Beyond this, future work should focus on developing delineation frameworks that balance statistical optimality with practical feasibility; advancing hybrid zoning approaches that integrate static soil-based structures with dynamic crop and climate variability; improving data robustness through systematic anomaly detection and multi-source integration; and translating complex methodologies into scalable, accessible decision-support systems. Finally, extending MZ applications toward crop rotation systems, sustainability objectives, and emerging AI-driven, context-aware frameworks remains an important direction for achieving broader real-world impact.

## Supplementary Materials

The following supporting information can be downloaded at: https://www.mdpi.com/article/10.3390/s26103249/s1, Table S1: PRISMA 2020 Checklist; File S1: Full screening decision dataset (containing human and AI model outputs, confidence scores, and rationales); File S2: Machine-readable review database exports (CSV and JSON formats with documentation); Database S1: A searchable web-based supplementary database of the 137 reviewed papers is accessible at https://roghiheidari.github.io/mz-delineation-review-code/ (accessed on 10 May 2026); The full computational code and the archived dataset are also available via the project’s public repositories [43,49].

## Abbreviations

The following abbreviations are used in this manuscript:

ANOVA	Analysis of Variance
BIC	Bayesian Information Criterion
CEC	Cation Exchange Capacity
CHI	Calinski–Harabasz Index
CWSI	Crop Water Stress Index
DEM	Digital Elevation Model
ECa	Apparent Electrical Conductivity
EDA	Estimation of Distribution Algorithms
EGI	Excess Green Index
EMI	Electromagnetic Induction
ET	Evapotranspiration
FCM	Fuzzy c-Means
FI	Fragmentation Index
FNEA	Fractal Net Evolution Approach
FPI	Fuzziness Performance Index
GNSS	Global Navigation Satellite Systems
GMM	Gaussian Mixture Model
HSS	Heidke Skill Score
ICLS	Integrated Crop–Livestock Systems
ICVI	Improved Cluster Validation Index
ILP	Integer Linear Programming
IoT	Internet-of-Things
JCR	Journal Citation Reports
LLM	Large Language Model
MAE	Mean Absolute Error
MLM	Mixed Linear Models
MPE	Modified Partition Entropy
MZ(s)	Management Zone(s)
NCE	Normalized Classification Entropy
NDRE	Normalized Difference Red Edge
NDVI	Normalized Difference Vegetation Index
NIR	Near-Infrared
OBIA	Object-Based Image Analysis
PCA	Principal Component Analysis
PoMZSA	Percentage of Management Zones Spatial Agreement
PSS	Proximal Soil Sensing
RMSE	Root Mean Square Error
SAR	Synthetic Aperture Radar
SDSS	Spatial Decision Support Systems
SI	Smoothness Index
SOM	Soil Organic Matter
SPR	Soil Penetration Resistance
TPI	Topographic Position Index
UAV	Unmanned Aerial Vehicle
VI/VIs	Vegetation Index/Vegetation Indices
VR	Variance Reduction

## Appendix A

**Table A1:** Reports excluded after full-text screening (*n* = 17) with reasons.

Paper ID	Title	Reason (Category)	Reason Details
1	Decoding local terroir: Data mining to predict sensory profiles of coffee beverage [166]	Not management-zone delineation (out of scope)	The study classifies land according to coffee beverage typicity and cup-quality profiles rather than delineating operational management zones.
22	Spatial Variability and Management Zones: Leveraging Geostatistics and Fuzzy Clustering [167]	Not field-scale/regional-scale study	The analysis covers a 3530 km^2^ catchment in the Mashhad Plain of northeast Iran.
37	Efficiency of Geostatistical Approach for Mapping and Modeling Soil Site-Specific Management Zones for Sustainable Agriculture Management in Drylands [168]	Not field-scale/regional-scale study	The study covers 1739.75 hectares (over 17 km^2^) in El-Ismaillia Governorate, Egypt.
53	Delineation of high-resolution soil carbon management zones using digital soil mapping: A step towards mitigating climate change in the Northeastern Himalayas, India [169]	Not field-scale/regional-scale study	The mapping was conducted across 46,624 hectares in the Sepahijala district of Tripura within a climate-change mitigation context.
56	Evaluation of Variable Application Rate of Fertilizers Based on Site-Specific Management Zones for Winter Wheat in Small-Scale Farming [170]	Insufficient methodological detail for MZ delineation	The study applies village-scale zoning across 458 smallholder fields (209 hectares total) rather than within a single continuous field and reports only two broad zones.
71	Vegetation indices as a Tool for Mapping Sugarcane Management Zones [171]	Not field-scale/regional-scale study	The study covers a sugarcane production area of approximately 140,000 hectares, subdivided into about 5500 fields across a regional landscape.
99	Decomposition-based heuristic for the zoning and crop planning problem with adjacency constraints [172]	Not management-zone delineation (out of scope)	This is a computer science optimization study evaluated on synthetic instances, with emphasis on computational performance rather than applied field-scale MZ delineation.
122	An integrated approach for the rectangular delineation of management zones and the crop planning problems [173]	Not management-zone delineation (out of scope)	The study develops an algorithmic framework and evaluates it on synthetic matrices rather than on an applied precision agriculture field experiment.
132	Soil mapping and delineation of management zones in the Western Ghats of coastal India [174]	Not field-scale/regional-scale study	The mapping was conducted across North Goa District, spanning approximately 1736 km^2^.
137	Evaluation of spatial distribution and regional zone delineation for micronutrients in a semiarid Deccan Plateau Region of India [175]	Not field-scale/regional-scale study	The study area comprises 11.48 million hectares.
141	Spatial variability of soil properties and delineation of soil management zones of oil palm plantations grown in a hot and humid tropical region of southern India [176]	Not field-scale/regional-scale study	The analysis spans two mandals (sub-districts), with soil samples collected at 1–2 km spacing.
151	Spatial Distribution and Management Zones for Sulphur and Micronutrients in Shiwalik Himalayan Region of India [177]	Not field-scale/regional-scale study	The study area covers approximately 53,483 km^2^.
162	Delineation of soil management zones for a rice cultivated area in eastern India using fuzzy clustering [178]	Not field-scale/regional-scale study	The study was conducted across Rajnagar Block in Kendrapada District, including nearly 255 inhabited villages.
180	Delineation of site specific nutrient management zones for a paddy cultivated area based on soil fertility using fuzzy clustering [179]	Not field-scale/regional-scale study	The research covers approximately 24,000 hectares of paddy fields in Someh-Sara county.
248	Delineation of Soil Management Zone Maps at the Regional Scale Using Machine Learning [180]	Not field-scale/regional-scale study	The study covers Bajestan county, spanning approximately 172,419 hectares.
249	Implications of Spatial Variability of Soil Physical Attributes in Delineating Site-Specific Irrigation Management Zones for Rice Crop [181]	Not field-scale/regional-scale study	The analysis covers paddy fields across Shaft and Fouman counties, totaling approximately 280 km^2^.
291	Establishing management zones of soil sulfur and micronutrients for sustainable crop production [182]	Not field-scale/regional-scale study	The study spans approximately 97,410 km^2^ across three states.

## Appendix B

**XML extraction rules and prompt (study-level extraction)**

This document contains the rules and response format used to extract structured, study-level information from full-text papers during the review workflow.

**Task**

You are assisting in analyzing research papers for a systematic review.

Your job is to determine whether each paper is relevant, and if it is, produce a strictly formatted XML summary using short, keyword-style entries (not descriptive sentences).

If the paper is not relevant, output an ‘exclude: …’ line explaining why.

**Inclusion criteria (all must be true)**

1. Field-scale agricultural context
  - Real crop fields (not greenhouse, pots, purely lab, urban, or forestry).
  - Focus on crop production or soil/water/nutrient/crop management.
2. Applying a zoning method that produces discrete management zones
  - Paper delineates sub-areas inside a field (management zones, site-specific management units, homogeneous zones, productivity zones, etc.),
  - using measured/sensed field data (soil, yield, EC, NDVI/RS, UAV, etc.).
3. Direct link to site-specific management decisions
  - Zones are explicitly intended to guide field operations, e.g., variable-rate:
  - variable-rate fertilizer, lime/gypsum, irrigation, seeding, pesticides, or similar,
  - or clear zone-specific agronomic recommendations (different input levels or field practices per zone).
  - Conceptual/tool papers are included only if they clearly target such within-field input decisions in real crop fields.
4. Original field study
  - Includes empirical field data and actual MZ delineation (not only theory, generic simulation, or pure review/meta-analysis).

**Exclusion criteria (any one is sufficient)**

Exclude (do NOT generate XML) if any of the following is true:

- No field-scale MZ/no within-field partitioning
- Only continuous mapping or classification (e.g., LAI, yield, soil properties, land cover) without defining management zones or within-field decision units.
- Wrong scale or context
- Only regional/landscape/farm-level planning zones with no clear field-scale MZs.
- Non-agricultural contexts (urban, forest, basins) unless clearly framed as crop-field management zones.
- Experimental setup not comparable
- Greenhouse/micro-plots/pots/lysimeters without spatial zoning of real fields.
- Pure method/theory without real field MZs
- Algorithms or optimization methods tested only on synthetic or generic examples, with no clear application to field-scale agricultural MZs.
- Quality/terroir zoning without agronomic input decisions
- Zoning used mainly for product or quality differentiation (wine quality, coffee beverage typicity, terroir branding, certification lots)
- and not clearly used to change within-field agronomic inputs (fertilizer, irrigation, seeding, etc.).
- This includes cases where zones guide only harvest grouping, drying strategies, storage, marketing, or labelling.

**Response format**

If excluded, return only one line:

exclude: <short reason>

Examples:

- ‘exclude: zoning focuses on coffee beverage quality/terroir, not agronomic inputs or VRT’
- ‘exclude: only continuous yield mapping, no management zones or site-specific decisions’
- ‘exclude: regional zoning, no field-scale management zones’

If included, return exactly one XML block:

**XML**

<Paper id=“NNN”>

<Column>Country</Column><Information>…</Information>

<Column>FieldSize</Column><Information>…</Information>

<Column>Data used for MZ</Column><Information>…</Information>

<Column>Auxilary data</Column><Information>…</Information>

<Column>Data used for validation</Column><Information>…</Information>

<Column>Sensors/DataSources names</Column><Information>…</Information>

<Column>SamplingDensity</Column><Information>…</Information>

<Column>Resolution</Column><Information>…</Information>

<Column>Methods</Column><Information>…</Information>

<Column>Sub-Methods-Internal</Column><Information>…</Information>

<Column>Zones</Column><Information>…</Information>

<Column>ManagementFocus</Column><Information>…</Information>

<Column>Crops</Column><Information>…</Information>

<Column>Validation Method</Column><Information>…</Information>

<Column>Notes</Column><Information>…</Information>

</Paper>

Use these exact column names (spelling and spaces):

- Country
- FieldSize
- Data used for MZ
- Auxiliary data
- Data used for validation
- Sensors/DataSources names
- SamplingDensity
- Resolution
- Methods
- Sub-Methods-Internal
- Zones
- ManagementFocus
- Crops
- Validation Method
- Notes

**Content style (Information values)**

- Each ‘Information’ value should be short.
- Prefer keywords or compact phrases (lists separated by ‘;’).
- Aim for under 300 characters per ‘Information’.
- Capture only the most important variables, methods, data sources, zones, and main findings.

**Plain text only**

- No HTML (no ‘<sup>’, ‘<sub>’, ‘<b>’, etc.).
- Write ‘R2’ instead of ‘$R^{2}$’.
- Use ‘p<0.05’ (or ‘p &lt; 0.05’ if needed inside XML).

**XML validity rules**

- Always follow the pattern:
- ‘<Column>ColumnName</Column><Information>…</Information>’
- Every ‘<Column>’ must have a matching ‘</Column>’.
- Every ‘<Information>’ must have a matching ‘</Information>’.
- No extra ‘</Column>’ or ‘</Information>’ tags.
- No text before ‘<Paper>’ or after ‘</Paper>’.
- If you need a ‘<’ inside text, write it as ‘&lt;’.

**Model query convention**

The user will ask, e.g.,

“Generate XML result for paper XXX.pdf based on rules.docx”

The model must respond with either:

- A single ‘<Paper>…</Paper>’ XML block (for included papers),

or

- A single line ‘exclude: <reason>’ (for excluded papers).

No extra explanations, comments, or markdown.

**No citation brackets**

In ‘Information’ values, do not include reference-style citations such as ‘[1]’, ‘[2–4]’, ‘[6, 18–21]’, etc.

Write the content (keywords or short phrases) without these bracketed reference numbers.
